# Mechanistic insights and *in vivo* HIV suppression by the BRD4-targeting small molecule ZL0580

**DOI:** 10.1371/journal.ppat.1013449

**Published:** 2026-02-02

**Authors:** Naveen Kumar, Zonghui Ma, Fuquan Long, Srinivasa Reddy Bonam, Hsien-Tsung Lai, Shwu-Yuan Wu, Haiying Chen, Nicholas C. Hazell, Jiani Bei, Xuefeng Liu, Yeqing Chen, Zhi Wei, Cheng-Ming Chiang, Jia Zhou, Haitao Hu

**Affiliations:** 1 Department of Microbiology and Immunology, University of Texas Medical Branch, Galveston, Texas, United States of America; 2 Chemical Biology Program, Department of Pharmacology and Toxicology, University of Texas Medical Branch, Galveston, Texas, United States of America; 3 Department of Sexually Transmitted Disease, Center of Infectious Skin Disease, Shanghai Skin Disease Hospital, School of Medicine, Tongji University, Shanghai, China; 4 CSIR-Indian Institute of Chemical Technology (IICT), Hyderabad, India; 5 Simmons Comprehensive Cancer Center, Department of Biochemistry, and Department of Pharmacology, University of Texas Southwestern Medical Center, Dallas, Texas United States of America; 6 Department of Pathology, The Ohio State University College of Medicine, Columbus, Ohio, United States of America; 7 Department of Computer Science, New Jersey Institute of Technology, Newark, New Jersey, United States of America; 8 Institute for Human Infections and Immunity, University of Texas Medical Branch, Galveston, Texas, United States of America; Katholieke Universiteit LeuvenKatholieke Universiteit LeuvenKatholieke Universiteit LeuvenKatholieke Universiteit LeuvenKatholieke Universiteit LeuvenKatholieke Universiteit LeuvenKatholieke Universiteit LeuvenKatholieke Universiteit LeuvenKatholieke Universiteit LeuvenKatholieke Universiteit Leuven, BELGIUM

## Abstract

Epigenetic suppression and durable silencing of HIV represent a promising strategy to achieve ART-free remission, consistent with the “block and lock” HIV cure paradigm. BRD4 is a host epigenetic reader and plays a critical role in HIV transcriptional regulation. We previously identified ZL0580, a first-in-class BRD4-selective small molecule distinct from the pan-BET inhibitor JQ1, which induces HIV epigenetic suppression. However, detailed molecular mechanisms, pharmacokinetics (PK), and *in vivo* HIV-suppressive efficacy of ZL0580 remain undefined. Here, we show that ZL0580 selectively targets BRD4 bromodomain 1 (BD1) through interaction with a key glutamic acid residue (E151), as determined by structural modeling and mutagenesis. Transcriptomic profiling by RNA-seq reveals that ZL0580 and JQ1 induce opposing gene expression programs, consistent with their distinct effects on HIV proviral transcription and latency. In a humanized mouse model of HIV infection, ZL0580 monotherapy, or in combination with ART, potently suppressed active HIV replication, reducing the plasma viremia to nearly undetectable levels, and delayed viral rebound following treatment interruption. Collectively, these findings establish ZL0580 as an epigenetic suppressor of HIV *in vivo* and provide proof-of-concept for its potential as a “block and lock” HIV cure candidate, warranting further optimization and development.

## Author summary

Current HIV therapies effectively suppress viral replication but do not eradicate the virus, necessitating lifelong treatment. A promising strategy for achieving durable remission is the “block and lock” approach, which aims to silence HIV through epigenetic regulation. Our previous work identified ZL0580 as a first-in-class small molecule that specifically targets BRD4 to induce epigenetic suppression of HIV. In the present study, we investigated the molecular mechanism and *in vivo* activity of ZL0580. Molecular docking and mutagenesis analyses revealed that ZL0580 selectively engages bromodomain 1 (BD1) of BRD4 through interaction with a critical glutamic acid residue (E151). Using a humanized mouse model of chronic HIV infection, we further demonstrate that ZL0580 reduces viral replication and delays viral rebound following treatment interruption *in vivo*. Together, these findings provide proof-of-concept for ZL0580 as a potential “block and lock” agent in vivo and support further development of epigenetic strategies aimed at achieving sustained, ART-free control of HIV.

## Introduction

Human immunodeficiency virus (HIV) persists in latent reservoirs that evade immune surveillance and remains refractory to antiretroviral therapy (ART), posing a major barrier to an HIV cure [1–3]. Although ART effectively suppresses active viral replication and reduces plasma viremia to undetectable levels [4], it does not eradicate latent reservoirs, which are

the primary source of viral persistence [5–7]. Consequently, treatment interruption almost invariably results in rapid viral rebound [3,8,9] and intensification of ART is ineffective in reducing reservoir size or residual viral transcription [10]. These limitations highlight the need for novel therapeutic strategies aimed at achieving HIV remission or cure [5,11].

HIV proviral transcription and latency are tightly controlled by host epigenetic and transcriptional mechanisms [12–14]. Unlike the “shock and kill” strategy, which seeks to reverse latency and eliminate infected cells [15–17], the “block-and-lock” approach aims to achieve a functional cure by reinforcing latency and preventing viral reactivation through modulation of host and viral pathways [18–20]. For examples, LEDGIN, an HIV integrase inhibitor, hampers viral transcription and prevents viral reactivation by retargeting the provirus to transcriptionally inactive sites [21–23]. The Tat inhibitor didehydro-cortistatin A (dCA) suppresses Tat-dependent transactivation and promotes deep latency by inducing epigenetic repression in the HIV promoter [24–26]. In HIV-infected humanized mouse models, dCA treatment reduced residual viral replication and delayed viral rebound following treatment interruption [27].

BRD4 is a host cell epigenetic reader belonging to the bromodomain (BD) and extra-terminal (ET) domain protein family (BET) [28,29]. It is characterized by two conserved tandem bromodomains (BD1 and BD2) and an ET domain [30–33]. A primary function of BRD4 is to act as a scaffold by binding to acetylated histones and recruiting partner proteins to the gene promoters, thereby modulating gene expression [34]. The role of BRD4 in regulating HIV transcription and latency has been documented [30,35–37]. Inhibition of BET/BRD4 by the pan-BET inhibitor JQ1 has been shown to activate HIV transcription [35–37], and JQ1 exhibits synergistic effects in reversing HIV latency when combined with other latency-reversing agents [38–40]. However, evidence from the studies of our group and others suggests that BRD4 has versatile function and exerts context-dependent effects on HIV transcription, largely determined by its specific interactions with histones and partner proteins [41,42].

In our previous studies, we identified ZL0580, a first-in-class BRD4-selective small molecule distinct from the pan-BET inhibitor JQ1, which induces HIV epigenetic suppression and promotes deeper state of latency in cellular models [41,43,44]. This effect has been demonstrated in multiple *in vitro* and *ex vivo* models, including J-Lat cells, primary CD4 ⁺ T cells, and peripheral blood mononuclear cells (PBMCs) from ART-suppressed individuals, as well as myeloid cells such as microglia and macrophages [41,43,44]. ZL0580 suppresses HIV by altering chromatin structure at the viral LTR and inhibiting Tat-mediated transcription elongation [41,43,44]. The HIV-suppressive effect of ZL0580 was recently confirmed by another study [23]. Despite these findings, the molecular basis of the selective interaction of ZL0580 and BRD4 (as compared with JQ1), its pharmacokinetics (PK), and *in vivo* HIV-suppressive activity (block-and-lock potential) remain undefined.

In this study, we addressed several gaps critical for advancing ZL0580 toward further development. First, molecular docking and protein mutagenesis identified the glutamic acid at position 151 in the BD1 of BRD4 as a key residue that mediates the selective ZL0580-BRD4 interaction. Second, RNA-seq analysis revealed that ZL0580 and JQ1 induce largely opposing transcriptomic profiles, consistent with their distinct effects on HIV transcription and latency. Third, we characterized *in vivo* PK and safety profiles of ZL0580 in rodents, which, together with its HIV-suppressive activity demonstrated in diverse *in vitro* and *ex vivo* models, support its therapeutic potential. Finally, in a humanized mouse model of HIV infection, we demonstrate that ZL0580 monotherapy, or its combination with ART, suppresses active HIV replication, reducing plasma viremia to nearly undetectable levels after two weeks of treatment, and also delays viral rebound after treatment interruption, providing early proof-of-concept evidence for its *in vivo* block-and-lock activity.

## Results

### Distinct binding modes of ZL0580 *vs*. (+)-JQ1 to human BRD4 BD1 domain

**Fig 1:**
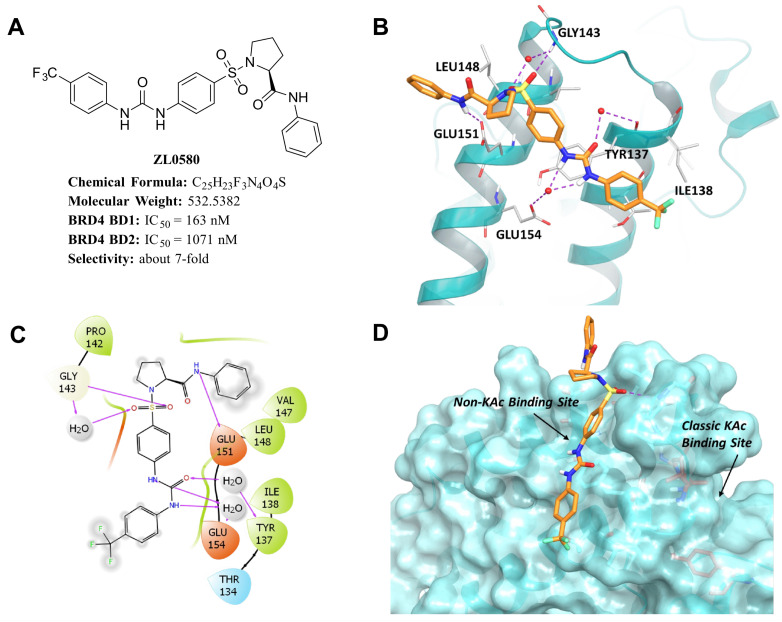
Predicted interactions between ZL0580 and BRD4 BD1. **(A)** Chemical structure of ZL0580 and its *in vitro* binding affinities toward BRD4 BD1 and BD2 domains. **(B)** Ribbon representation showing ZL0580 (orange stick) docked into the non-KAc binding site of BRD4 BD1 (based on PDB ID: 6U0D), forming strong hydrogen bond interactions (purple dotted lines) with key residues Glu151, Glu154, Tyr137, Gly143, and Leu148. **(C)** 2D interaction diagram illustrating the hydrogen bonding interactions (purple arrows) between ZL0580 and residues at the new binding site of BRD4 BD1. **(D)** Surface representation of BRD4 BD1 showing the binding of ZL0580 at the non-KAc binding site, highlighting its spatial orientation and interaction surface.

ZL0580 was identified as a selective BRD4 BD1 inhibitor in our previous studies (Fig 1A) [41]. To elucidate its molecular interactions with BRD4 BD1, we performed molecular docking study based on the co-crystal structure of BRD4 BD1 in complex with ZL0590, a close analog of ZL0580 [45]. The co-crystal structure of ZL0590 complexed with BRD4 BD1 was used for molecular simulation study to explore the key interactions between ZL0580 and BRD4 BD1 [45]. Docking results revealed that ZL0580 adopts a binding mode similar to ZL0590. As shown in Fig 1B-1C, ZL0580 forms several direct and water-mediated indirect hydrogen bond interactions with key residues in BRD4 BD1 protein, which are predicted to contribute to the high binding affinity and selectivity of ZL0580 for BRD4 BD1. Specifically, ZL0580 forms two water-mediated hydrogen bonds with the carbonyl group of GLU154 residue through two NH groups of the urea moiety. Moreover, the carbonyl oxygen of the urea moiety forms a water-mediated hydrogen bond with the hydroxyl group of TYR137. Two oxygen atoms of the sulfonyl group of ZL0580 form direct and water-mediated hydrogen bond interactions with the NH group of GLY143 residue, respectively. Additionally, the NH group of the amide moiety forms a direct hydrogen bond with the carbonyl oxygen atom of GLU151 residue (Fig 1B-1C). Unlike JQ1, ZL0580 targets a novel binding site spatially distinct from the classical acetyl-lysine (KAc) binding pocket occupied by (+)-JQ1 (Fig 1D). Collectively, the docking results indicate the high binding affinity and selectivity of ZL0580 for BRD4 BD1 at this noncanonical binding site, distinct from the classical KAc binding pocket occupied by JQ1.

### Mutagenesis identifies key residue mediating ZL0580 binding to BRD4 BD1

**Fig 2:**
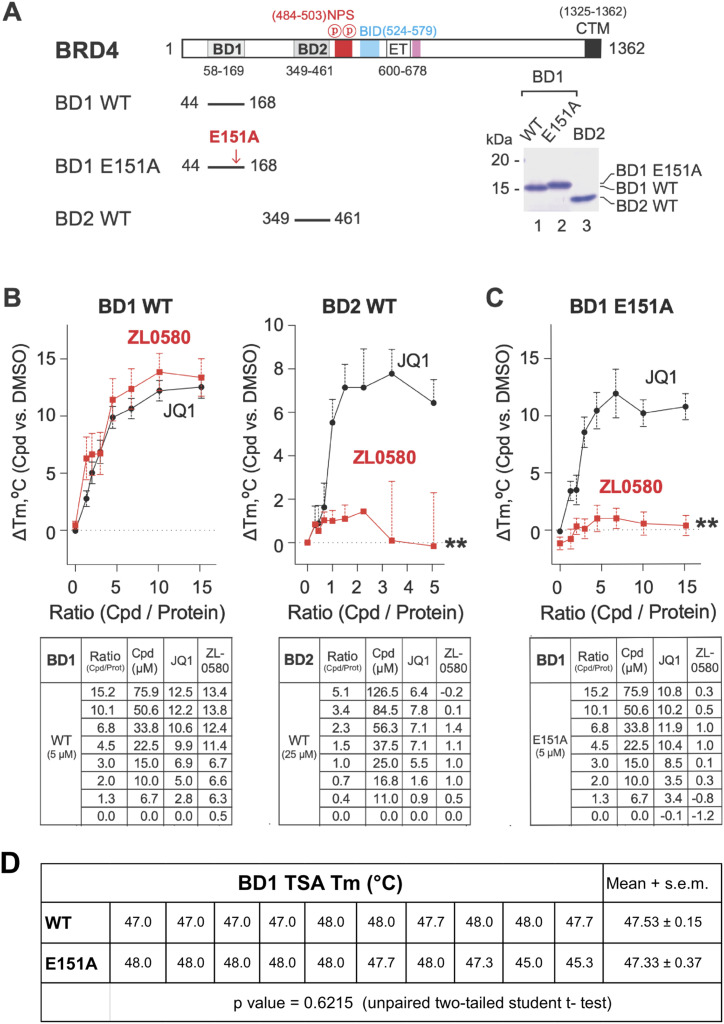
Mutagenesis identifies key residues for selective binding of ZL0580 to BRD4 BD1. **(A)** Schematic representation of BRD4 domain organization of full-length BRD4, indicating the positions of bromodomains (BD1, BD2), nuclear protein recognition motif (NPS), extra-terminal domain (ET), and C-terminal motif (CTM), with numbers denoting amino acid residues. Constructs used for mutagenesis, including wild-type BD1 (BD1 WT), BD1 with an E151A mutation (BD1 E151A), and wild-type BD2 (BD2 WT), where the red arrow indicates the position of the E151A mutation in BD1. SDS-PAGE analysis showing the expression and purity of recombinant BD1 WT, BD1 E151A, and BD2 WT proteins, with molecular weights (kDa) indicated. **(B)** Thermal stabilization of BRD4 bromodomains by JQ1 and ZL0580 is shown through differential scanning fluorimetry (DSF) curves, depicting the change in melting temperature (ΔTm) of BD1 WT (left) and BD2 WT (right) upon titration with increasing concentrations of JQ1 (black circles) and ZL0580 (red squares). The table below each figure shows compound/protein (cpd/prot) Ratio (column 2), compound concentrations (column 3), ΔTm for JQ1 (column 4), ΔTm for ZL0580 (column 5). Data is presented as mean ± SEM, normalized to DMSO control. **(C)** The effect of E151A mutation on ZL0580 binding to BRD4 BD1 is further illustrated by a DSF curve showing the ΔTm of BD1 E151A upon titration with JQ1 (black circles) and ZL0580 (red squares). Data is presented as mean ± SEM, normalized to DMSO control. ** p < 0.01 (ZL0580 compared to JQ1 in B-C). **(D)** TSA melting temperatures (Tm) for BD1 WT and the E151A variant. Individual measurements are shown. Mean ± SEM: WT, 47.53 ± 0.15°C; E151A, 47.33 ± 0.37°C. No significant difference was detected (unpaired two-tailed Student’s t test, p = 0.6215).

To confirm that ZL0580 targets a novel binding site distinct from the classical KAc binding pocket of BRD4 BD1 occupied by (+)-JQ1, we performed mutagenesis of key residues located at the new binding site in BRD4 BD1 that was predicted by molecular docking. Docking analysis indicated that GLU151 residue with a polar side chain forms a critical hydrogen bond with the amide NH group ZL0580 (Fig 1). Therefore, GLU151 was selected for mutagenesis, consistent with our previous study demonstrating that ZL0590 binds to a similar noncanonical binding site as revealed by its co-crystal structure with BRD4 BD1 (PDB ID: 6U0D) [45]. The BRD4 BD1 mutant, in which GLU151 was substituted with alanine (E151A), was expressed and purified for binding analysis with ZL0580 or JQ1 as compared to the wild-type (WT) BRD4 BD1 (Fig 2A).

To investigate the interactions between ZL0580 and the BRD4 bromodomains (BD1 and BD2), we performed thermal shift assay (TSA) to assess the effects of ZL0580 on BRD4 BD stability, using (+)-JQ1 as a positive control and DMSO as a vehicle control. WT BD1 (5 µM), WT BD2 (25 µM), and the BD1 E151A mutant (5 µM) were treated with ZL0580 or (+)-JQ1 across eight increasing concentrations (compound/protein ratio) (Fig 2B-2C). The melting temperatures shifts (ΔTm) were measured and the ΔTm curves as a function of compound-to-protein ratios were generated (Fig 2B–2C). The results showed that ZL0580 dose-dependently increased the stability of WT BD1 but not BD2, while (+)-JQ1 dose-dependently increased the stability of both WT BD1 and BD2, suggesting that ZL0580 selectively binds to BD1 sparing BD2, which is distinct from (+)-JQ1, as a pan-BET inhibitor. The TSA results is consistent with our previously reported binding affinities of these compounds with BET proteins measured by time-resolved fluorescence energy transfer (TR-FRET) assays [41,45]. Notably, ZL0580 showed almost no effect on the stability of BD1 E151A mutant, while (+)-JQ1 retained the dose-dependent effect on E151A mutant (Fig 2C). The results suggest that GLU151 is critical for the binding of ZL0580 to BRD4 BD1, consistent with the docking results (Fig 1B-1D), which is distinct from the classical “KAc” pocket on BD1 recognized by (+)-JQ1. This surface-exposed binding site may enable ZL0580 to more efficiently modulate critical protein-protein interactions (PPIs) between BRD4 and its partner proteins [41]. As an additional control, the Tm of WT BD1 and E151A mutant in the absence of compound was also measured by TSA. The mean Tm of 10 independent measurements was compared using an unpaired two-tailed Student’s t-test (p = 0.6215) (Fig 2D). The WT BD1 and E151A mutant exhibited nearly identical mean Tm - 47.53 ± 0.15 °C and 47.33 ± 0.37 °C, respectively, indicating that the E151A mutation itself has a negligible effect on BD1 protein stability.

### Opposing transcriptomic profiles induced by ZL0580 and JQ1

**Fig 3:**
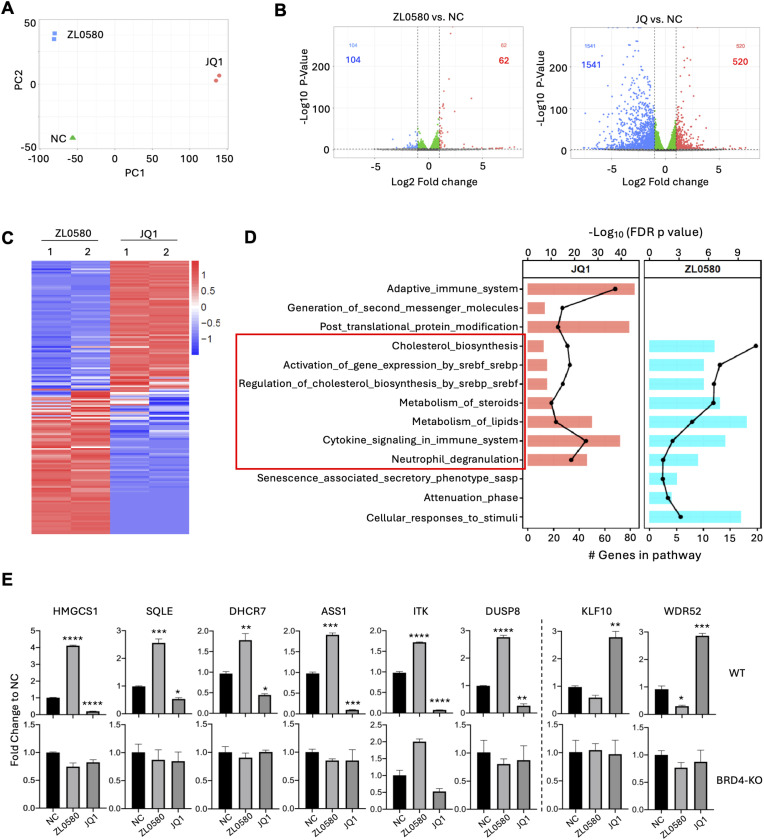
Transcriptomic profiling and pathway analysis of JQ1 and ZL0580 treatments. J-Lat cells (clone 10.6) were treated with ZL0580 (5 μM), JQ1 (5 μ **M)**, or with DMSO as the negative control (NC). 24 hours after treatment, RNA was extracted from cells for sequencing analysis. RNA-Seq was performed in duplicate for each condition. **(A)** Principal Component Analysis (PCA) plot showing distinct clustering of samples treated with ZL0580, JQ1, and NC, indicating differential global gene expression profiles. **(B)** Differential gene expression analysis with volcano plots between ZL0580 vs. NC (left) and JQ1 vs. NC (right). Red dots indicate significantly upregulated genes, blue dots indicate significantly downregulated genes, and green dots represent non-significant genes. The number of upregulated and downregulated genes is shown in red and blue, respectively. Cutoff: p < 0.001 and log2 fold change > 1. **(C)** Heatmap representing the differentially expressed genes and the expression patterns of genes significantly regulated by ZL0580 and JQ1 treatments relative to NC. Red represents upregulation and blue represents downregulation. Samples from each condition are shown in duplicates. **(D)** Gene-set enrichment analysis (GSEA) shows a bar graph of biological processes significantly enriched among differentially expressed genes in JQ1 (left, red) and ZL0580 (right, blue) treatments. The length of the bars indicates the number of overlapping genes. The black line plot superimposed on each bar graph represents the log10 (FDR-adjusted p-value). The pathways shared by the two compounds were highlighted by a red box. **(E)** Quantitative RT-qPCR validation of selected differentially expressed genes (DEGs). Each bar represents the fold change in expression relative to NC for ZL0580 and JQ1 treated samples, after normalization to GPADH. Data is shown as mean ± SEM from three replicates. Statistical comparison was performed using one-way ANNOVA. * p < 0.05; ** p < 0.01; *** p < 0.001; **** p < 0.0001.

Our previous studies showed that although ZL0580 and JQ1 both target BRD4, they exert opposing effects on HIV proviral transcription and latency [41,44]. To gain further mechanistic insights and assess their global transcriptomic impact, we performed RNA-Seq analysis on latently HIV-infected J-Lat cells (clone 10.6) [46], the same clone used in our previous study for compound screening and mechanistic investigations [41]. Cells were treated with ZL0580 (5 μM), JQ1 (5 μM), or with DMSO as the negative control (NC). At this concentration, both compounds induced notable impacts on HIV transcription while minimizing potential toxicity [41]. 24 hours after treatment, RNA was extracted from cells for sequencing analysis. RNA-Seq was conducted on duplicate samples for each condition. Principal component analysis (PCA) demonstrated that ZL0580 induced a distinct but less pronounced transcriptional shift relative to NC compared to JQ1 (Figs 3A and A in S1 Text). Differential gene expression analysis supported this observation (Fig 3B): ZL0580 significantly regulated 166 genes (62 upregulated and 104 downregulated), whereas JQ1 affected 2,061 genes (520 upregulated and 1,541 downregulated) using a threshold of p < 0.001 and log2 fold change > 1 (Fig 3B). A similar finding was observed when using a threshold of p < 0.001 and log2 fold change > 0.5 (Fig B in S1 Text). The narrower transcriptomic impact of ZL0580 likely reflects its selective modulation of BRD4, in contrast to JQ1, which targets all four BET family members [47].

Analysis of differentially expressed genes revealed largely inverse transcriptomic profiles induced by ZL0580 and JQ1. Heatmap analysis showed that almost all genes downregulated by ZL0580 were upregulated by JQ1, and vice versa (Fig 3C), consistent with their opposing effects on HIV transcription and latency [41,44]. Gene Set Enrichment Analysis (GSEA) showed that both compounds regulate similar biological pathways, with 7 of the top 10 enriched pathways shared between the two compounds, including cholesterol biosynthesis, metabolism of steroid, metabolism of lipids, activation of gene expression by SREBP (Sterol Regulatory Element-Binding Proteins), cytokine signaling, and neutrophil degranulation (Fig 3D). Further analysis indicated that JQ1 also strongly regulated pathway of the adaptive immune system, whereas ZL0580 had minimal or no effect on this pathway (Fig 3D and 3C in S1 Text). This observation aligns with our previous finding that ZL0580 minimally affects immune activation and cytokine production in human T cells and PBMCs [41]. Collectively, these results indicate that ZL0580 induces more selective and less extensive transcriptomic changes than JQ1, and that the two compounds trigger largely opposing gene expression programs in J-Lat cells.

To validate the RNA-Seq results, RT-qPCR analysis was performed on a subset of differentially expressed genes (DEGs) (Fig 3E). Eight representative DEGs were selected based on statistical significance (adjusted p values) and their involvement in key pathways identified by GSEA, including lipid metabolism/cholesterol biosynthesis (*HMGCS1*, *SQLE*, *DHCR7*) and cytokine signaling (*ITK* and *DUSP8*). Relative gene expression levels were normalized to the housekeeping gene GAPDH. In WT J-Lat cells, ZL0580 upregulated genes such as *HMGCS1*, *SQLE*, *DHCR7*, *ASS1*, *ITK,* and *DUSP8*, whereas these genes were downregulated by JQ1 relative to NC. Conversely, *KLF10* and *WDR52* were downregulated by ZL0580 but upregulated by JQ1, consistent with the RNA-Seq results (Fig 3E, top). As an additional control, RT-qPCR was also performed on RNA isolated from BRD4-knockout (KO) J-Lat cells that received the same treatments. The generation and characterization of BRD4-KO J-Lat cells were described previously [41]. Notably, the contrasting regulatory effects of ZL0580 and JQ1 on these representative DEGs were largely abolished in the absence of BRD4 (Fig 3E; bottom), further supporting that BRD4 is the primary target mediating the distinct transcriptomic effects.

### Effect of ZL0580 and JQ1 on p-TEFb recruitment to the HIV promoter

Using ChIP-qPCR, our previous study examined Tat recruitment to the HIV promoter in J-Lat cells (clone 10.6) and showed that JQ1 increased, whereas ZL0580 decreased, Tat binding, consistent with their opposing effects on Tat-mediated transactivation and transcription elongation [41]. To directly assess the impact of ZL0580 and JQ1 on p-TEFb recruitment, J-Lat cells (clone 10.6) were treated with DMSO (negative control, NC), PMA, PMA + ZL0580 (5 μM), or PMA + JQ1 (5 μM). Twenty-four hours later, cells were subjected to ChIP-qPCR using an anti-CDK9 antibody, with isotype IgG as the ChIP control.

**Fig 4:**
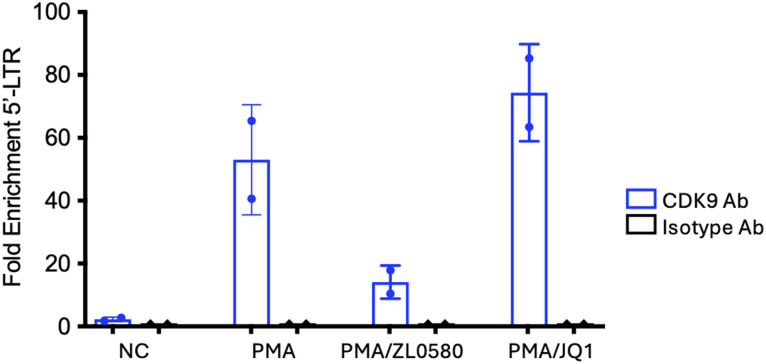
ChIP-qPCR measurement of CDK9 binding to HIV 5′-LTR. J-Lat cells (10.6) were treated with DMSO (negative control, NC), PMA (50ng/ml), PMA + ZL0580 (5 μM), or PMA + JQ1 (5 μM) as indicated. 24 hours after treatments, ChIP-qPCR was performed using a CDK9-specific antibody to examine the recruitment of CDK9 to HIV 5’-LTR in J-Lat cells. ChIP using an isotype IgG was included for normalization. Data were shown as fold change in the precipitated HIV 5′ LTR by the CDK9 antibody relative to the control isotype IgG for each treatment condition.

PMA stimulation led to a substantial increase in CDK9 occupancy at the HIV promoter (~19-fold relative to NC) (Fig 4). Relative to PMA alone, JQ1 further enhanced CDK9 recruitment (~1.3-fold), whereas ZL0580 markedly reduced CDK9 binding (~3.8-fold) (Fig 4). As a control, the input HIV 5’-LTR DNA in the sheared chromatin prior to ChIP was comparable across the four treatment groups (Fig D in S1 Text). These findings are consistent with the previously observed effects on Tat recruitment and support that ZL0580 suppresses HIV transcription, at least in part, by limiting p-TEFb–mediated HIV transcription elongation [41].

### *In vivo* pharmacokinetics and toxicity profile of ZL0580 in mice

**Fig 5:**
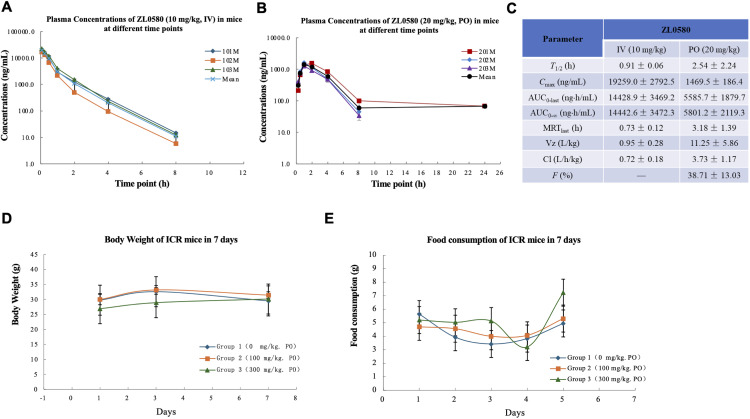
*In vivo* pharmacokinetics (PK) and toxicity profiles of ZL0580 in ICR mice. **(A)** Plasma concentrations of ZL0580 following intravenous administration at 10 mg/kg in male ICR mice (n = 3 per group) measured at various time points. **(B)** Plasma concentrations of ZL0580 following oral administration at 20 mg/kg in male ICR mice (n = 3) over time. **(C)** Summary of *in vivo* PK parameters of ZL0580 in male ICR mice. **(D)** Body weight changes in ICR mice over the treatment period (n = 10 per group). **(E)** Food consumption changes in ICR mice monitored throughout the study (n = 10 per group).

Given the favorable *in vitro* target engagement and HIV-suppressive activity of ZL0580, we next evaluated *in vivo* pharmacokinetic (PK) properties and safety profile of the compound in mice prior to efficacy testing. In ICR mice, intravenous administration of ZL0580 (IV, 10 mg/kg) resulted in a favorable PK profile, characterized by high *C*_max_ and AUC values (*C*_max_ = 19259.0 ± 2792.5 ng/mL; AUC_0-last_ = 14428.9 ± 3469.2 ng/mL; AUC_0-∞_ = 14442.6 ± 3472.3 ng/mL) (Fig 5A, 5C). Oral administration of ZL0580 (20 mg/kg) showed an acceptable moderate bioavailability (*F* = 38.71 ± 13.03%) (Fig 5B, 5C).

Toxicity assessments in mice revealed that ZL0580 was well tolerated *in vivo* (Fig 5D-5E). Daily administration at doses of 0 mg/kg, 100 mg/kg, or 300 mg/kg for 7 days caused little to no changes in body weights (Fig 5D) or food consumption (Fig 5E). No clinical signs of toxicity were observed throughout the study (Table A in S1 Text). Serum biochemistry as well as necropsy and gross pathological examinations revealed no abnormalities at the end of the study (Tables B and C in S1 Text). Collectively, these findings demonstrate that ZL0580 has favorable PK properties and an acceptable safety profile in mice, supporting its further evaluation in HIV-suppression studies *in vivo*.

### *In vivo* activity of ZL0580 to suppressing HIV in HIV-infected humanized mice

To assess *in vivo* HIV-suppressive activity of ZL0580, we utilized a humanized mouse model (Hu-mice) generated by engrafting human CD34 + hematopoietic stem cells (HSCs) into the immunodeficient NOD SCID gamma (NSG) mice (the Jackson Laboratory). Twelve Hu-mice, aged 16–20 weeks and engrafted with human HSCs from a single donor, were enrolled in the study. Prior to HIV infection, human immune cell reconstitution was evaluated in all animals. Human CD45 + cells were detected in the peripheral blood of all 12 Hu-mice, ranging from 36% to 62% with a median of 45% (Fig E(A) in S1 Text). Further characterization revealed successful reconstitution of major human immune cell subsets (Fig E(B) in S1 Text). Within the human CD45 + population, the median proportions of CD3 + T cells, CD19 + B cells, and CD33 + myeloid cells were 14.9%, 74.3%, and 3.7%, respectively, confirming robust human hematopoietic reconstitution (Fig E(B) in S1 Text).

**Fig 6:**
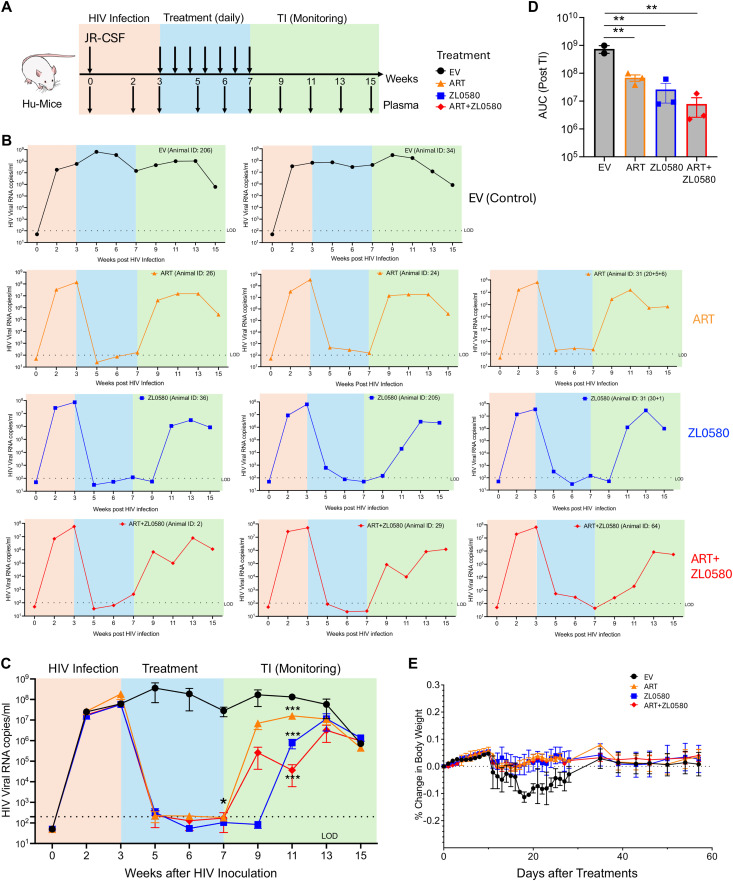
*In vivo* activities of treatments to suppress HIV in humanized mice. **(A)** Experimental design and timeline for the *in vivo* study using HIV-infected CD34 + Hu-mice. At week 0, 12 Hu-mice were intravenously inoculated with a single dose of HIV-1 JR-CSF (10 ng HIV p24 antigen per animal). Plasma samples were collected at weeks 2 and 3 after HIV inoculation for confirmation of HIV infection by RT-qPCR and HIV p24 ELISA. At week 3, 11 out of the 12 Hu-mice were confirmed with systemic HIV infection (the one without infection was removed from the study). The 11 Hu-mice with successful HIV infection were randomized into four treatment groups at week 3: Empty Vehicle (EV) (n = 2), ART (n = 3), ZL0580 (n = 3), and ART + ZL0580 (n = 3). Treatment regimens were initiated at week 3 and administered daily by intraperitoneal until week 7. Plasma viral loads were closely monitored during the treatments at weeks 5, 6, 7. From week 7, all animals were subjected to treatment interruption (TI) and plasma viral loads were closely monitored once every 2 weeks until week 15. **(B)** Kinetics of plasma viral loads (HIV RNA copies/ml) post HIV inoculation for each mouse in EV (black circles), ART (orange triangles), ZL0580 (blue squares), and ART + ZL0580 (red diamonds) treatment groups. The shaded areas denote the phase of establishing systemic HIV infection (orange), treatment phase (blue), and treatment interruption (TI) monitoring phase (green). RT-qPCR was performed in duplicate and the mean of viral RNA copies for each sample at different time points was shown. **(C)** Summary data for kinetics of plasma viral loads over weeks post HIV inoculation for all animals in the four treatment groups: EV (black circles), ART (orange triangles), ZL0580 (blue squares), and ART + ZL0580 (red diamonds). Data are presented as mean ± SEM. Statistical comparison of viral loads among the four groups at each time point was performed using one-way ANNOVA. * at week 7 and *** at week 13 denote significant difference of the individual treatment group (ART, ZL0580, or ART + ZL0580) as compared to the EV group. The dashed line indicates the limit of detection (LOD). **(D)** Comparison of area under the curve (AUC) among the four groups after treatment interruption (TI) between weeks 7 and 15. Statistical comparison was performed using one-way ANNOVA. **(E)** Percentage change in body weight over days after treatment for all treatment groups. Data are presented as mean ± SEM. In this figure: * p < 0.05; ** p < 0.01; *** p < 0.001.

Next, at week 0, all12 Hu-mice were intravenously inoculated with a single dose of HIV-1 JR-CSF (equivalent to 10 ng HIV p24 antigen per animal) (Fig 6A). Systemic HIV infection was confirmed in 11 out of the 12 mice at weeks 2 and 3 after HIV inoculation, based on the detection of plasma viral RNAs by a single-step RT-qPCR (Fig 6B-6C). Systemic HIV infection in the Hu-mice was also confirmed by ELISA quantification of plasma HIV p24 antigens at weeks 2 and 3 after HIV inoculation (Fig F in S1 Text). The 11 Hu-mice with successful HIV infection were then randomized into four treatment groups at week 3: empty vehicle control (EV) (n = 2), ART (n = 3), ZL0580 (n = 3), and ART + ZL0580 (n = 3). Drug formulations included: ART (Raltegravir 1.2 mg/mouse, Emtricitabine 3.2 mg/mouse, Tenofovir 3.2 mg/mouse), ZL0580 (3.8 mg/mouse), and a combination of both ART + ZL0580 (same concentration of individual compounds). All drugs were formulated in the buffer consisting of 10% DMSO, 10% Solutol HS 15, and 80% HP-β-CD (20%, w/v). The empty vehicle (EV) group received only the formulation buffer. Treatment regimens were initiated at week 3 and administered daily by intraperitoneal injection (Fig 6A). Plasma viral loads were closely monitored during the treatments.

The results showed that in the EV control group, plasma viral loads were consistently high throughout the study in both animals (Fig 6B-6C). In contrast, all three treatment groups (ART alone, ZL0580 alone, or ART + ZL0580) demonstrated drastic reductions in plasma viremia. By week 5 (after two weeks of daily treatment), the plasma viral loads were reduced to nearly undetectable levels in all mice of all the three groups, including the ZL0580 monotherapy group (Fig 6B), providing strong evidence that ZL0580 alone as a host-directed compound robustly suppresses active HIV replication *in vivo*. The low-to-undetectable HIV viremia in plasma persisted through weeks 7, during which all mice remained under treatments in the three groups (Fig 6B). At week 7, The mean ± SEM viral copies of EV, ART, ZL0580, ART + ZL0580 group were 2.84 × 10^7^ ± 1.38 × 10^7^, 182 ± 23, 105 ± 28, and 173 ± 139, respectively. The differences in viral loads for the three treated groups compared to the EV control group were statistically significant with an effect size of 0.77 (R^2^ by one-way ANOVA) and p < 0.05 for EV vs. ART, EV vs. ZL0580, and EV vs. ART + ZL0580 (Fig 6C).

To assess viral rebound following treatment cessation, an analytical treatment interruption (TI) was initiated at week 7 across all the groups (Fig 6A). Plasma viral loads were subsequently measured once every two weeks from week 7. At week 9 (two weeks post-TI), rapid viral rebounds were observed in all three Hu-mice in the ART group (mean viral copies: 6.96 × 10^6^ copies/mL), despite the levels remained markedly lower than the EV group (mean viral copies: 1.75 × 10⁸ copies/mL) (Fig 6C). Notably, all mice in the ZL0580 alone group maintained undetectable viremia at week 9 (Fig 6C), suggesting that compared to ART, ZL0580 alone could delay viral rebounds. However, viremia was detectable at week 9 in the ART + ZL0580 group (mean viral copies: 2.59 × 10^5^ copies/ml), although levels were substantially lower than the ART only group. At week 11 (four weeks post-TI), plasma viremia became detectable in all mice in both ZL0580 and ART + ZL0580 groups; however, viral loads in these two groups at this time point remained markedly lower than those in the ART group: mean viral RNA copies of 7.7 × 10⁵ copies/mL in ZL0580 group and of 3.6 × 10⁴ copies/mL in ART + ZL0580 group, as compared to mean viral RNA copies of 1.6 × 10⁷ copies/mL in ART group. Statistical analysis showed that, at week 11, the plasma viral loads in all three treated groups were significantly lower than that in the EV group (Fig 6C). By week 15 (eight weeks post-ATI), viral loads in all the treatment groups converged to similar levels (Fig 6C). To more quantitatively assess the impact of different treatments on viral rebounds after TI, we quantified area under the curve (AUC) between weeks 7 and 15 (Fig 6D), which combines the parameters of time (x-axis) and viral loads (y-axis) after TI. The data indicated that, compared to the EV control, all three treatments (ART, ZL0580, and ART + ZL0580) significantly suppressed viral rebounds, with the combination group (ART + ZL0580) exhibiting a trend towards the most pronounced effect (Fig 6D).

To assess general health of the mice and potential protective effects of the treatments, body weights of Hu-mice were closely monitored. As shown in Fig 6E, all treatment groups, including those receiving ART, ZL0580, or combined ART + ZL0580, maintained stable body weights with no significant deviations throughout the study. There was a slight reduction in body weights of Hu-mice in the EV group following HIV infection. However, no statistically significant difference was detected as compared to the three treatment groups (Fig 6E). Nonetheless, these data demonstrate that daily intraperitoneal administration of ZL0580 alone, or in combination with ART, is well tolerated in Hu-mice over the course of treatment. These results provide *in vivo* evidence that ZL0580 robustly suppresses active HIV replication, reducing plasma viral loads to nearly undetectable levels, and modestly delays viral rebound following treatment interruption.

### HIV DNA in blood cells across different groups

**Fig 7:**
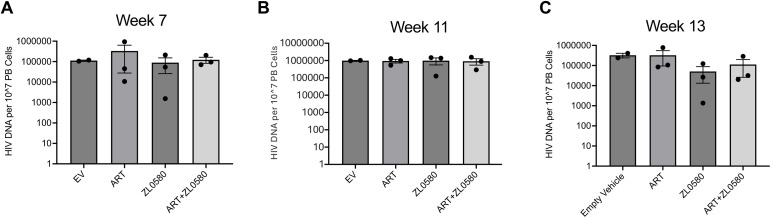
HIV DNA copies in blood cells. HIV DNA copies per 10^7^ peripheral blood (PB) cells at week 7 **(A)**, week 11 **(B)**, and week 13 (C) for Empty Vehicle (EV), ART, ZL0580, and ART + ZL0580 treatment groups. Data are presented as mean ± SEM, with individual data points shown.

To assess whether ZL0580 or ART affects HIV DNA levels, we quantified cell-associated HIV DNA in the peripheral blood of Hu-mice after treatments. Blood cells collected at weeks 7, 11 and 13 were subjected to DNA extraction and quantification of HIV DNA by qPCR. At week 7, no significant differences in HIV DNA copies were detected among the EV control, ART, ZL0580, or ART + ZL0580 groups (Fig 7A). We also measured HIV DNA at week 11, the time point at which ZL0580 and ZL0580 + ART showed the strongest suppression of plasma viremia. The data showed comparable HIV DNA levels among all four groups at this time point as well (Fig 7B). By week 13, HIV DNA levels also remained similar across all groups (Fig 7C). This data indicates that while ZL0580 and ART robustly suppress active viral replication (as reflected by plasma viral RNA levels), neither treatment significantly alters the levels of HIV DNA in peripheral blood of Hu-mice.

## Discussion

The “block and lock” strategy represents a promising approach for achieving a functional HIV cure [18–20]. In this study, we evaluated bioavailability and *in vivo* HIV-suppressive activity of the BRD4-targeting small molecule ZL0580 in animal models. ZL0580 was bioavailable and exhibited a favorable safety profile, with no evident toxicity in mice. In a humanized mouse model, ZL0580 monotherapy potently suppressed active HIV replication and modestly delayed viral rebound after treatment cessation. To our knowledge, this is the first demonstration of robust *in vivo* HIV suppression by a small-molecule compound targeting host epigenetic machinery. Further, we identified key residues mediating the selective interaction of ZL0580 with BRD4 BD1, providing new mechanistic insights into its distinct modulation of HIV transcription and latency compared with the pan-BET inhibitor JQ1.

Our previous studies demonstrated that ZL0580 and JQ1 exhibit distinct binding profiles within the BET protein family, with ZL0580 selectively targeting BRD4 BD1, whereas JQ1 non-selectively binds both BD1 and BD2 of all BET proteins [41,43,44]. The molecular basis underlying this selectivity was unclear. The present study identified the glutamic acid 151 (E151) in BRD4 BD1 as a key residue mediating selective ZL0580-BRD4 interaction. This finding corroborates previous structural evidence supporting BRD4 BD1 as a druggable site [48] and confirms that ZL0580 engages a distinct binding interface, avoiding the conserved acetyl-lysine (KAc) binding pocket targeted by pan-BET inhibitors such as (+)-JQ1 [41,44,49]. The selective targeting of BD1 by ZL0580, and its preferential engagement of BRD4 over other BET proteins, confers a pharmacological profile that may minimize disruption of global transcriptional networks, a limitation of the first-generation BET inhibitors [44,45,49].

RNA-seq analysis of ZL0580- and JQ1-treated cells revealed several interesting results. First, modulation of BRD4 by ZL0580 or JQ1 results in both upregulation and downregulation of gene expression. This is consistent with previous studies reporting that BRD4 inhibition - via either genetic knockout [50] or pharmacologic inhibition [51] - can activate or repress transcription in a gene-specific manner, highlighting the dual role of BRD4 as a transcriptional activator [52] or repressor [53]. Second, while JQ1 modulated the expression of a broad array of cellular genes (>4000), ZL0580 elicited a more selective and attenuated transcriptional response, affecting approximately 760 genes. This is likely attributable to the selective targeting of BRD4 by ZL0580, in contrast to JQ1’s pan-BET inhibition profile [47]. The restrained transcriptional footprint of ZL0580 supports a notion that selective BD1 inhibition may allow for more targeted repression of viral transcription while sparing host gene regulation critical for homeostasis [54]. Third, gene enrichment analysis reveals that multiple pathways were commonly regulated by both compounds but the gene expression within these pathways were inversely regulated. This aligns with their opposing effects on HIV transcription [41,43,44] and reinforces the idea that that both compounds modulate overlapping molecular targets through distinct mechanisms. Lastly, ZL0580 induced minimal to changes in immune and pro-inflammatory gene expression pathways, corroborating previous finding that ZL0580 does not perturb immune activation and cytokine production in human T cells and PBMCs [41]. This contrasts with the widespread transcriptional regulation of immune-related pathways by JQ1, which has been linked to cytokine release and immunomodulatory effects [55,56].

In the humanized mouse model of HIV infection, ZL0580 monotherapy robustly suppressed active HIV replication and reduced plasma viremia to nearly undetectable levels after two weeks of daily treatment in Hu-mice. This *in vivo* result is highly consistent with the *in vitro* and *ex vivo* evidence reported in our previous studies using cell line and primary cell models [41,43,44]. Mechanistically, by inhibiting Tat-mediated recruitment of positive transcription elongation factor b (p-TEFb) and preventing transcriptional elongation, ZL0580 induces a transcriptionally inert state at the HIV long terminal repeat (LTR) [41]. ChIP-qPCR analysis in the current study further showed that JQ1 increased CDK9 recruitment to the HIV promoter, whereas ZL0580 decreased CDK9 binding, consistent with their previously reported opposing effects on Tat recruitment [41]. This HIV-suppressive mechanism of ZL0580 is further reinforced by its ability to stabilize a repressive chromatin structure at the proviral promoter [41]. In this Hu-mouse model of HIV infection, ZL0580 alone delayed viral rebound following TI compared to ART alone, despite the effect was modest (~2 weeks). Interestingly, viremia became detectable two weeks after TI in the combination ART + ZL0580 group, although levels remaining substantially lower than the ART-only group. The underlying reason for this observation is unclear, but it may reflect interference of ZL0580’s potent repressive activity in the presence of additional ART drugs (e.g., altered cellular uptake) or limited sample size of the animal study. Nonetheless, following viral rebounds, the plasma viremia in the ZL0580 and ZL0580 + ART groups remained markedly lower than those in the ART only group. These findings together provide early *in vivo* proof-of-concept evidence for ZL0580 as a potential HIV “block and lock” candidate. However, the lack of durable viral suppression by ZL0580 after treatment cessation highlights a limitation of the compound, which is likely attributed to its relatively short half-life. Future research should be conducted to enhance the half-life and durability of HIV suppression by this class of molecules. Additionally, combining multiple “block and lock” agents with distinct modes of action is also a promising approach to achieve more durable HIV suppression after treatment cessation.

Indeed, the latency-promoting potential of ZL0580 is enhanced when used in combination with LEDGINs, as demonstrated by Pellaers et al. [23]. LEDGINs function by retargeting HIV provirus into transcriptionally inactive genomic regions, thereby minimizing the likelihood of proviral reactivation [21,22]. The synergistic effects between ZL0580 and LEDGINs support a potential combinatorial “block-and-lock” strategy aimed at enforcing deep and durable latency [23]. This dual-targeting approach addresses both integration site bias and the maintenance of transcriptional repression of the integrated provirus. Previous studies have shown that LEDGINs promote integration into genomic regions less permissive to transcriptional reactivation [22,57]. Nevertheless, residual low-level expression may persist from certain integration sites, highlighting the need to combine LEDGINs with epigenetic silencers such as ZL0580. The complementary mechanisms of these latency-promoting agents (LPAs) highlight the importance of concurrently targeting both the chromatin context and transcriptional machinery to achieve durable HIV silencing.

Given the duration and complexity of humanized mouse studies involving chronic HIV infection and continuous drug administrations, a limitation of the present study is the small number of animals used, which constrains overall statistical power. Although the data showed a clear delay in viral rebound by ZL0580 and the low inter-animal variability within each group allowed for the detection of statistically significant differences in plasma viremia between the three treatment groups and the EV control at weeks 7 and 11, the study is not sufficiently powered to examine statistical significance among the three treatment groups. Nonetheless, these proof-of-concept findings warrant further confirmation and validation in larger-scale animal studies and non-human primate (NHP) models. Additionally, the present study assessed only the acute toxicity profile of ZL0580 in animals. The long-term tolerability of ZL0580 following repeated, chronic dosing remains unknown and warrants evaluation in future studies.

In summary, our study supports that ZL0580 is a BD1-selective BRD4 modulator with mechanistic and functional distinctions from the classical pan-BET inhibitors. Its ability to suppress HIV transcription, while minimizing global disruption of host chromatin architecture and immune activation, positions it as a valuable tool and therapeutic candidate for HIV latency-promoting strategies. Future efforts should be pursued to improve the half-life and durability of HIV suppression by this class of molecules. Additionally, combinatorial approaches incorporating multiple LPAs with distinct mechanisms-such as epigenetic silencers (ZL0580), LEDGINs, and Tat inhibitors - are likely necessary to achieve maximal and sustained HIV suppression or silencing.

## Materials and methods

### Ethics statement

The use of human CD34 + engrafted NSG mice (CD34 + Hu-mice) in this study was approved by the Institutional Animal Care & Use Committee (IACUC) (No.: 2105034) at the University of Texas Medical Branch (UTMB). The fully reconstituted CD34 + Hu-NSG mice were obtained from the Jackson Laboratory (Stock No: 005557). The human cells used in the study were de-identified and the study is considered as non-human subject research.

### Molecular docking studies

The molecular docking study was performed using the Schrödinger Small-Molecule Drug Discovery Suite. The crystal structure of compound ZL0590 in complex with BRD4 BD1 (PDB ID: 6U0D) was downloaded from RCSB PDB Bank and prepared with Protein Prepared Wizard. During protein preparation, hydrogens were added, crystal waters were removed while water molecules around the KAc pocket were maintained, and other parameters were used by default. The 3D structure of ZL0580 was generated with Schrödinger Maestro, and the initial lowest energy conformation was calculated with LigPrep. For the docking study, the grid center was chosen on the centroid of the KAc site occupied by ZL0590 in the cocrystal structure and a 24 × 24 × 24 Å grid box size was fixed. The docking was performed with Glide using the XP protocol. The docking pose was incorporated into Schrödinger Maestro for the ligand-receptor interaction analysis.

### Protein expression and purification

Plasmid construction and subsequent protein expression and purification of BRD4 proteins were performed using OverExpress C43(DE3) bacterial competent cells (Lucigen) as described in our previous studies [45,58]. In brief, 12 L of bacterial cultures grown to OD600 between 0.6-0.8 were induced at 37°C for 4 h by 1 mM isopropyl β-bd-1-thiogalactopyranoside (IPTG). Bacterial sonication supernatant in the lysis buffer (500 mM NaCl, 20 mM Tris-HCl pH 7.9 at 4°C, 20% glycerol, 1 mM EDTA, 0.2 mM DTT, and protease inhibitors) was incubated with glutathione (GSH) agarose beads (GOLDBIO, G-250–100) overnight with rotation at 4°C. The protein-bound GSH beads were washed three times with the lysis buffer and digested with human rhinovirus 3C protease (HRV-P3C; Thermal Fisher 88946) at 4°C for 4 h, according to the manufacture’s procedure, to remove GST tag from BD1/2 proteins. Untagged BRD4-BD1 and -BD2 were separated from HRV-P3C via passing through a HiTrap Q HP anion exchange chromatography column (GE Healthcare, GE17-1154-01) and eluted by a salt gradient from 100 mM to 1 M NaCl. The peak fractions were combined and concentrated using Amicon Ultra Centrifugal Filter with a molecular weight cut-off of 3 kDa (Millipore, UFC900324) in the final buffer 10 mM HEPES pH 7.5 and 150 mM NaCl.

### Thermal Shift Assay (TSA)

TSA experiments were performed in triplicate in a 384-well plate (Bio-Rad, HSP3801) using the CFX384 Real-Time C1000 touch thermal cycler (Bio-Rad) as described previously [45,58]. In each 10-μL reaction, 5 μM of WT and mutant BD1 and 25 μM of BD2 was mixed with 2 μL of 25X Sypro Orange protein dye (Invitrogen, S6650) in 20 mM HEPES pH 6.5, 100 mM NaCl, and 2.5% glycerol with 2 μL of ZL580 or JQ1 in 5% DMSO (final 0–126.5 μM). Fluorescence signals generated by dye binding to unfolded proteins were monitored by using the melt curve protocol at the rate of 1°C temperature increment in 30 s from 25 °C to 95 °C with signal capture every 1 °C. The melt curves obtained by fluorescence signal (F) versus temperature (T) were converted to melting peaks using Bio-Rad CFX Manager v3.1 software. Tm is the temperature at which 50% of protein is unfolded and bound by the dye. ΔTm is the difference of Tm comparing compound-treated vs. untreated (i.e., DMSO-only) samples.

### RNA-sequencing

J-Lat cells (Clone 10.6) were treated with 5 µM of either JQ1 or ZL0580, or with DMSO as negative control (NC), for 24 hours to assess the impact of these compounds on global gene expression. Following the treatment, total RNA was extracted from the cells using the RNeasy Mini Kit (Qiagen) in accordance with the manufacturer’s instructions. To ensure the integrity and quality of the isolated RNA, samples were first quantified and then analyzed by agarose gel electrophoresis. Clear and intact ribosomal RNA bands confirmed that the RNA was of high quality and suitable for downstream applications. The verified RNA samples were subsequently submitted for high-throughput RNA sequencing (RNA-seq) to evaluate transcriptomic changes induced by the respective treatments. RNA sample quality was assessed using Agilent Bioanalyzer and samples with a RIN value larger than 7.0 were used for library preparation. RNA-Seq libraries were prepared using NEBNext Poly(A) mRNA Magnetic Isolation module (E7490) and NEBNext Ultra II Directional RNA Library Prep kit for illumina (E7760) following manufacturer’s recommended procedure. The library quality was assessed using Agilent Bioanalyzer High Sensitivity DNA chip and real-time PCR and pooled together for sequencing. The pooled library was sequenced on Illumina NextSeq 550 Mid Output 150 cycle kit for Paired End 75 bp reads targeting ~20 million paired reads per sample. The sequencing reads were mapped to human reference genome hg38 using STAR v2.7.5c [59] using recommended ENCODE parameters. Differential gene expression analysis was performed using Bioconductor DESeq 2 package [60].

### RT-qPCR verification of cellular gene expression

RNA extracted from wild-type (WT) and BRD4-KO J-Lat cells (Clone 10.6) following different treatments were subjected to RT-qPCR to quantify the expression of selected cellular genes (*HMGCS1, SQLE, DHCR7, ASS1, ITK, DUSP8, KFL10, WDR52*). RT-qPCR was performed as previously reported [41]. Primer sequences for cellular genes are listed in the Table D in S1 Text.

### ChIP and qPCR

ChIP was performed using a method reported previously [41]. About 15 × 10⁶ J-Lat cells were treated according to the conditions described in the Main Text. After treatment, cells were collected, washed once with PBS, and resuspended in 10 mL PBS. To cross-link protein–DNA complexes, cells were fixed with 37% formaldehyde, and the reaction was quenched by adding 10Xglycine and incubating for 5 min at room temperature. The cells were then pelleted by centrifugation, and the pellets were incubated in ice-cold lysis buffer for 30 min. Nuclei were isolated by centrifugation at 5,000 rpm for 10 min and resuspended in 700 μL shearing buffer supplemented with protease inhibitors (PIC) and PMSF. Chromatin was fragmented by sonication. The resulting sheared chromatin was used to set up ChIP by adding the magnetic beads and 5 µg of anti-CDK9 (PA5149556; Thermo Fisher) or 5ug of rabbit IgG control (02–6102; Thermo Fisher). After extensive washing with the appropriate ChIP buffers, immune complexes were eluted, reverse cross-linked, and treated with proteinase K. DNA was purified using phenol–chloroform extraction and subsequently analyzed by qPCR. Primer sequences targeting the HIV 5′-LTR promoter are presented in the supplementary Table E in S1 Text. ChIP-qPCR results were quantified using the fold-enrichment method as previously reported [41].

### Pharmacokinetics (PK) studies

Animal experiments assessing PK and *in vivo* toxicity profile of ZL0580 was performed by the contracting research organization (CRO) Sundia. The studies were performed according to the NIH Guide for Care and Use of Experimental Animals. The PK studies of ZL0580 was performed using male ICR mice (18–22 g). The mice were randomly divided into two groups (*n* = 3). The mice of the group 1 were intravenously administrated with ZL0580 [10 mg/kg, in 10% DMSO + 10% Solutol HS 15 + 80% HP-β-CD (20%, w/v)] and the mice of the group 2 were orally administrated with ZL0580 [20 mg/kg, in 10% DMSO + 90% HP-β-CD (20%, w/v)]. Blood samples of group 1 and group 2 mice were collected at the indicated time points post treatment and put into heparinized centrifugation tubes, which were centrifuged under the condition of 6800 rpm and 4 °C for 6 min. The supernatant was collected and stored at -20 °C for further LC-MS analysis. Before the plasma concentrations (AUC) were determined, standard curves were generated with various concentrations of ZL0580 and the internal standard (IS). All PK parameters were calculated with WinNonlin 6.4 software.

### *In vivo* toxicity studies

The acute toxicity studies were performed in both male and female ICR mice. The mice were divided into 3 groups (each group, n = 10, 5 males and 5 females). Group 1 was the vehicle group without ZL0580 treatment (po, 20% DMA + 20% Solutol HS15 + 60% HP-β-CD (20%, w/v)). Group 2, and group 3 mice were orally administrated with ZL0580 at doses of 100 mg/kg and 300 mg/kg [in 20% Dimethyl Acetamide (DMA) + 20% Solutol HS15 + 60% HP-β-CD (20%, w/v)], respectively. The body weights of all mice were weighed on day 1, 3, and 7 after treatments. The mean body weights of all mice in each group were calculated, and the standard curves were generated with the mean body weights of each group mice at different recorded time points. The food consumption masses of each group mice were recorded on day 1, 2, 3, 4, and 5 after treatments. The mean food consumption of all mice in each group was calculated, and the standard curves were generated with the mean food consumption of each group at different recorded time points.

### RNA-sequencing

J-Lat cells (Clone 10.6) were treated with 5 µM of either JQ1 or ZL0580, or with DMSO as negative control (NC), for 24 hours to assess the impact of these compounds on global gene expression. Following the treatment, total RNA was extracted from the cells using the RNeasy Mini Kit (Qiagen) in accordance with the manufacturer’s instructions. To ensure the integrity and quality of the isolated RNA, samples were first quantified and then analyzed by agarose gel electrophoresis. Clear and intact ribosomal RNA bands confirmed that the RNA was of high quality and suitable for downstream applications. The verified RNA samples were subsequently submitted for high-throughput RNA sequencing (RNA-seq) to evaluate transcriptomic changes induced by the respective treatments. RNA sample quality was assessed using Agilent Bioanalyzer and samples with a RIN value larger than 7.0 were used for library preparation. RNA-Seq libraries were prepared using NEBNext Poly(A) mRNA Magnetic Isolation module (E7490) and NEBNext Ultra II Directional RNA Library Prep kit for illumina (E7760) following manufacturer’s recommended procedure. The library quality was assessed using Agilent Bioanalyzer High Sensitivity DNA chip and real-time PCR and pooled together for sequencing. The pooled library was sequenced on Illumina NextSeq 550 Mid Output 150 cycle kit for Paired End 75 bp reads targeting ~20 million paired reads per sample. The sequencing reads were mapped to human reference genome hg38 using STAR v2.7.5c [59] using recommended ENCODE parameters. Differential gene expression analysis was performed using Bioconductor DESeq 2 package [60]. The raw RNA-sequencing data were deposited to the Gene Expression Omnibus (GEO) with the accession number GSE315344.

### Compound formulation for mouse administration

All humanized mice used in this study weighed approximately 20 grams, and drug dosages were calculated accordingly. The following compounds were administered to mice daily: Raltegravir (Ral) at 60mg/kg (1.2 mg/mouse), Emtricitabine (Emt) at 160mg/kg (3.2 mg/mouse), Tenofovir (Ten) at 160mg/kg (3.2 mg/mouse), and ZL0580 at 160mg/kg (3.8 mg/mouse). ART drugs were purchased from MedChemExpress. ZL0580 was synthesized in-house as previously reported [41]. All compounds were dissolved in a formulation vehicle composed of 10% DMSO, 10% Solutol HS 15, and 80% hydroxypropyl-β-cyclodextrin (HP-β-CD, 20% w/v). Compounds were administered via intraperitoneal injection daily.

### Humanized mouse study

Mice were 16-week-old at the initiation of the experiments. All mice received human CD34 + stem cells of a single donor and human immune cell reconstitution was confirmed for all mice. A total of 12 Hu-mice were initially included in the study with each tagged and assigned with a unique ID. At week 0, all Hu-mice were intravenously (IV) infected with HIV-1 JR-CSF at a single dose of 10ng HIV p24 equivalent virus per animal. Blood/plasma viral loads were monitored and quantified weekly by a single-step RT-qPCR until systemic HIV infection was established. Following HIV exposure, one animal failed to develop systemic infection and was excluded from the cohort. At week 3, the remaining 11 Hu-mice were randomized into four groups for subsequent treatments: Empty Vehicle (EV) (n = 2), ART (n = 3), ZL0580 (n = 3), and ART + ZL0580 (n = 3). The control mice received a mixture of DMSO, Solutol HS 15, and hydroxypropyl-β-cyclodextrin (HP-β-CD, 20% w/v), which was considered the empty vehicle control (EV). ART and ZL0580 compounds were administered to mice via intraperitoneal injection daily for four weeks (from week 3 to week 7). Compound dosages were described above. Plasma samples were collected from mice either weekly or biweekly for quantification of viral loads (RNA copies). Blood cells were also collected at weeks 7 and 13 for quantification of cell-associated HIV DNA in blood. Mouse weights were monitored after treatment.

### HIV p24 ELISA

HIV p24 antigen levels in plasma from humanized mice were quantified using the HIV-1 p24 Antigen Capture Assay Kit (ZeptoMetrix Corporation, USA) according to the manufacturer’s instructions. Briefly, standards and appropriately diluted plasma samples were added to microplate wells pre-coated with anti-p24 antibodies and incubated at room temperature for 2 hours. After washing six times with wash buffer, 100 µL of detector antibody was added and incubated for 1 hour, followed by another six washes. Substrate solution (100 µL) was then added and incubated for 30 minutes at room temperature. Upon color development, 100 µL of stop solution was added, and absorbance was measured at 450 nm using a microplate reader.

### Plasma viral RNA quantification

Viral RNA was isolated from plasma samples using the QIAamp Viral RNA Mini Kit (Qiagen) according to the manufacturer’s instructions. Quantification of viral RNA was performed using the PrimeTime One-Step RT-qPCR Master Mix (IDT) on a CFX Connect Real-Time PCR System (Bio-Rad). Following RNA extraction, one-step RT-qPCR was carried out using LTR Gag-specific primers and a HEX-labeled probe. Forward: 5′-GATCTCTCGACGCAGGACTC-3′, Reverse: 5′-CGCTTAATACCGACGCTCTC-3′, Probe: 5’-/5HEX/CCAGTCGCC/ZEN/GCCCCTCGC CTC/3IABkFQ. Each 20 µL reaction mixture contained 10 µL of PrimeTime One-Step Master Mix, 1 µL of each primer, 1 µL of probe, and 2 µL of extracted RNA. The thermal cycling conditions were as follows: cDNA synthesis at 50 °C for 15 minutes, polymerase activation at 95 °C for 15 minutes, followed by 44 cycles of denaturation at 95 °C for 15 seconds and annealing/extension at 60 °C for 1 minute. All reactions were performed in duplicate. Following PCR amplification, Viral RNA copies were quantified using the established HIV Gag standard curve as reported in our previous studies [41,44].

### Quantification of HIV DNA in blood cells

Quantification of cell-associated HIV DNA in blood cells was performed by qPCR as previously described with modifications [61]. Blood cells were counted and subjected to DNA extraction using the Qiagen QIAamp DNA Blood Mini Kit (QIAGEN). qPCR primers and probe: Forward: 5′-GATCTCTCGACGCAGGACTC-3′, Reverse: 5′-CGCTTAATACCGACGCTCTC-3′, Probe: 5’-/5HEX/CCAGTCGCC/ZEN/GCC CCTCGCCTC/3IABkFQ. The thermal cycling conditions were as follows: 95 °C for 15 minutes, followed by 44 cycles of denaturation at 95 °C for 15 seconds and annealing/extension at 60 °C for 1 minute. All reactions were performed in duplicate. HIV DNA copies were quantified using the HIV Gag standard curve as described above, as well as in a previous study [41], and normalized to peripheral blood cell numbers.

### Statistics

Statistical analysis was performed using the GraphPad Prism. Data are presented as mean ± SEM where appropriate. Statistical comparison was performed using a non-paired Student’s t test for two groups and a one-way ANOVA for more than two groups. Two-tailed p values were denoted, and p values of less than 0.05 were considered significant.

## Supporting information

S1 Text Figures A-F and Tables A-E. (PDF)
