# Supplementary material for: Mechanistic insights and in vivo HIV suppression by the BRD4-targeting small molecule ZL0580
Source: PLoS Pathog. 2026 Feb 2;22(2):e1013449. doi: 10.1371/journal.ppat.1013449 (PMC12880750; doi:10.1371/journal.ppat.1013449)
Supplement: S1 Text — (PDF) [file ppat.1013449.s001.pdf]

Supplementary Information

Mechanistic insights and *in vivo* HIV suppression by the BRD4-targeting small molecule ZL0580

Naveen Kumar, et al.

Fig A

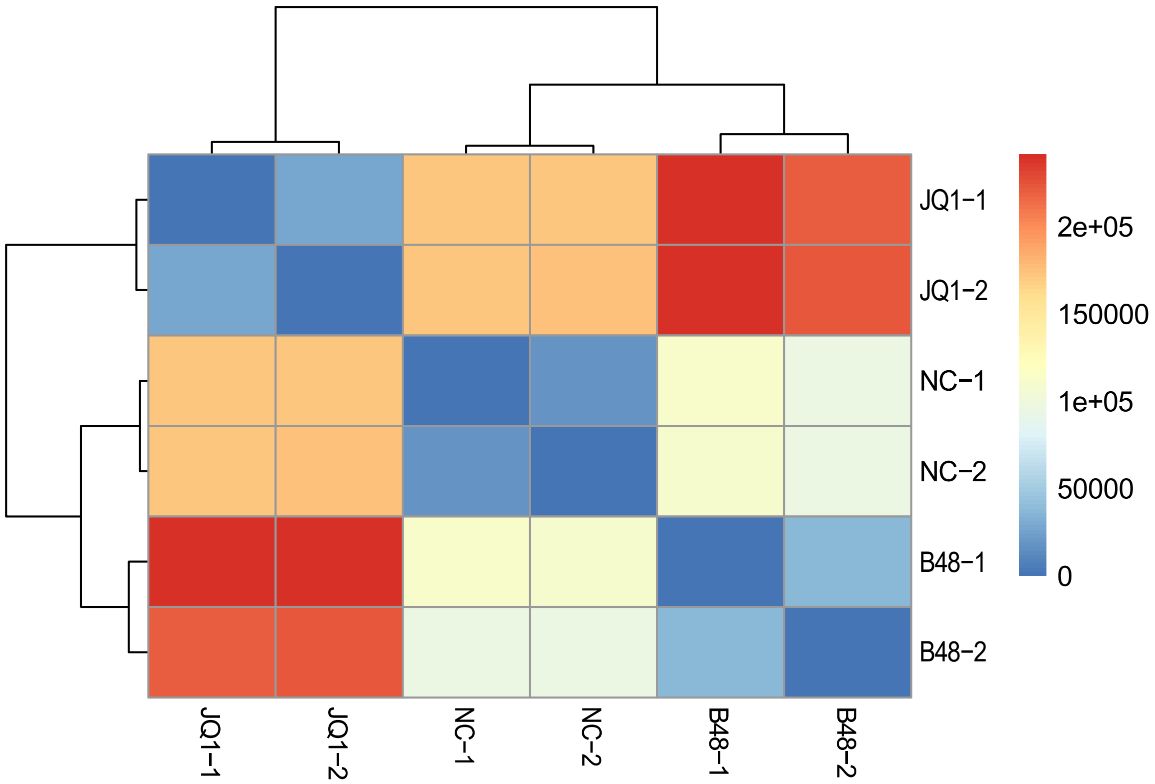

Fig A. Sample-to-sample distance heatmap of gene expression profiles. Heatmap illustrates the relationship between samples based on their gene expression profiles, with color intensity indicating the distance (or dissimilarity) between any two samples. Both rows and columns are arranged using hierarchical clustering, which groups samples with similar expression patterns closer together. Typically, samples belonging to the same experimental condition are expected to cluster together, reflecting their shared expression profiles.

Fig B

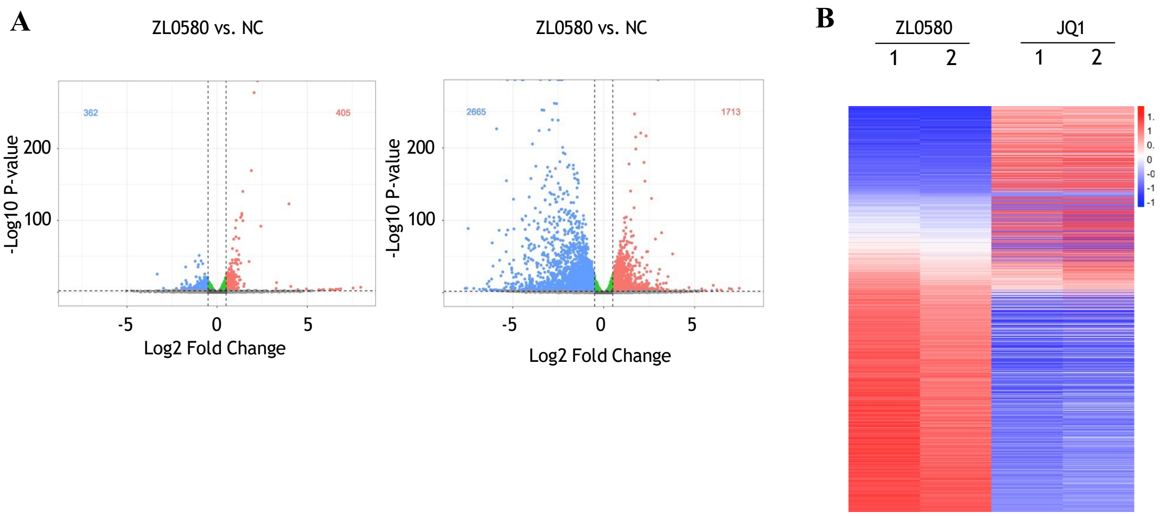

Fig B. Global differential gene expression and clustering analysis of ZL0580 and JQ1 treatments compared to negative control. **(A)** Differential gene expression analysis with volcano plots between ZL0580 vs. NC (left) and JQ1 vs. NC (right). Red dots indicate significantly upregulated genes, blue dots indicate significantly downregulated genes, and green dots represent non-significant genes. The number of upregulated and downregulated genes is shown in red and blue, respectively. Cutoff: $p < 0.001$ and log2 fold change > 0.5. **(B)** Heatmap representing the differentially expressed genes and the expression patterns of genes significantly regulated by ZL0580 and JQ1 treatments relative to NC. Red represents upregulation and blue represents downregulation. Samples from each condition are shown duplicates.

Fig C

A

Top Pathways Regulated by ZL0580 vs. NC		
**Gene Set Name**	**# Genes in Overlap (k)**	**FDR q-value**
REACTOME_CHOLESTEROL_BIOSYNTHESIS	12	1.78E-20
REACTOME_ACTIVATION_OF_GENE_EXPRESSION_BY_SREBF_SREBP	10	8.45E-14
REACTOME_REGULATION_OF_CHOLESTEROL_BIOSYNTHESIS_BY_SREBP_SREBF	10	1.08E-12
REACTOME_METABOLISM_OF_STEROIDS	13	1.38E-12
REACTOME_METABOLISM_OF_LIPIDS	18	1.29E-08
REACTOME_CYTOKINE_SIGNALING_IN_IMMUNE_SYSTEM	14	5.55E-05
REACTOME_NEUTROPHIL_DEGRANULATION	9	3.13E-03
REACTOME_SENESCENCE_ASSOCIATED_SECRETORY_PHENOTYPE_SASP	5	3.64E-03
REACTOME_ATTENUATION_PHASE	4	4.09E-04
REACTOME_CELLULAR_RESPONSES_TO_STIMULI	17	1.77E-06

B

Top Pathways Regulated by JQ1 vs. NC		
**Gene Set Name**	**# Genes in Overlap (k)**	**FDR q-value**
REACTOME_ADAPTIVE_IMMUNE_SYSTEM	83	1.82E-23
REACTOME_CHOLESTEROL_BIOSYNTHESIS	12	4.77E-11
REACTOME_ACTIVATION_OF_GENE_EXPRESSION_BY_SREBF_SREBP	15	1.17E-11
REACTOME_REGULATION_OF_CHOLESTEROL_BIOSYNTHESIS_BY_SREBP_SREBF	15	8.26E-10
REACTOME_METABOLISM_OF_STEROIDS	19	7.35E-07
REACTOME_METABOLISM_OF_LIPIDS	50	4.98E-08
REACTOME_CYTOKINE_SIGNALING_IN_IMMUNE_SYSTEM	72	7.49E-16
REACTOME_NEUTROPHIL_DEGRANULATION	46	5.46E-12
REACTOME_GENERATION_OF_SECOND_MESSENGER_MOLECULES	13	1.08E-09
REACTOME_POST_TRANSLATIONAL_PROTEIN_MODIFICATION	79	1.53E-08

Fig C. Pathways regulated by ZL0580 and JQ1. An enrichment analysis was performed using the online tool GSEA (https://www.gsea-msigdb.org/gsea/index.jsp) and showed the top 10 pathways significantly regulated by ZL0580 **(A)** and JQ1 **(B)** compared to a negative control. Differentially expressed genes were selected using a threshold of $p < 0.001$ and log2 fold change > 1. Pathways are ranked by FDR q-value. The number of genes overlapped in each gene set is also indicated.

Fig D

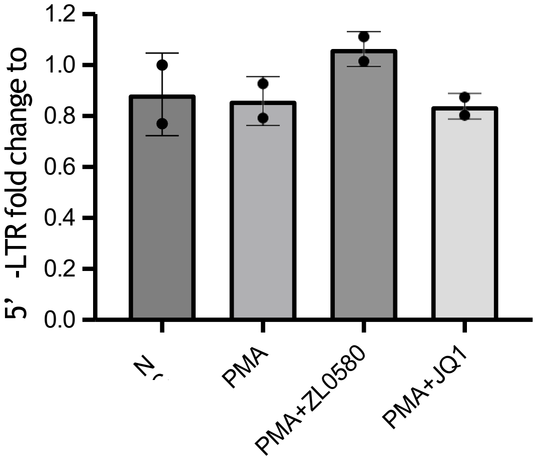

Fig D. Quantification of input HIV 5’-LTR by qPCR. Sheared chromatin prior to ChIP was subjected to qPCR quantification of input HIV 5’-LTR DNA (supplementary to Fig. 4). qPCR was performed in duplicate. The data is shown as fold change to DMSO treatment (negative control; NC).

Fig E

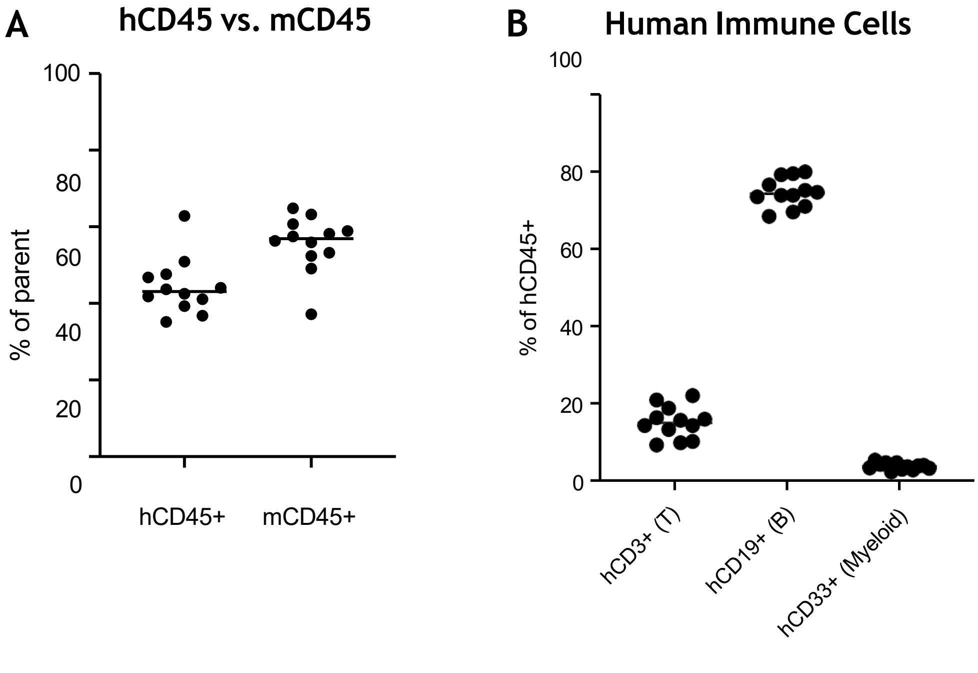

Fig E. Human immune cell reconstitution in humanized mice. (A) Percentage of human CD45+ (hCD45+) and mouse CD45+ (mCD45+) cells in blood. **(B)** Proportions of human CD3+ T cells, CD19+ B cells, and CD33+ myeloid cells within the hCD45+ population. Each data point represents an individual mouse, and horizontal lines indicate the mean.

Fig F

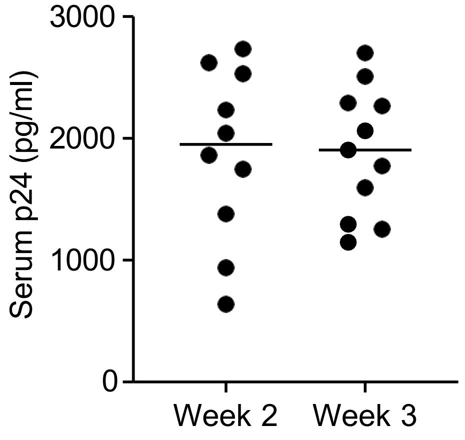

Fig F. Serum HIV p24 concentrations in individual humanized mice at weeks 2 and weeks 3 after HIV inoculation. Serum p24 concentration was quantified by ELISA.

Table A. The result of clinical observation after oral dose of ZL0580 at 100, 300 mg/kg dose levels.

Compound	Group (Dose level)	Gender	Animal No.	Dose volume（mL/kg）	volume（mL）	Detail Clinical Observation During Administration Period						
						Day 1(twice)	Day 2	Day 3	Day 4	Day 5	Day 6	Day 7
Vehicle	Group 1（0 mg/kg. PO）	Female	601/F	10	0.30	01	01	01	01	01	01	01
			602/F		0.32	01	01	01	01	01	01	01
			603/F		0.30	01	01	01	01	01	01	01
			604/F		0.32	01	01	01	01	01	01	01
			605/F		0.31	01	01	01	01	01	01	01
		Male	606/M		0.29	01	01	01	01	01	01	01
			607/M		0.27	01	01	01	01	01	01	01
			608/M		0.28	01	01	01	01	01	01	01
			609/M		0.30	01	01	01	01	01	01	01
			610/M		0.29	01	01	01	01	01	01	01
ZL0580	Group 2（100 mg/kg. PO）	Female	701/F	10	0.31	01	01	01	01	01	01	01
			702/F		0.32	01	01	01	01	01	01	01
			703/F		0.33	01	01	01	01	01	01	01
			704/F		0.32	01	01	01	01	01	01	01
			705/F		0.30	01	01	01	01	01	01	01
		Male	706/M		0.28	01	01	01	01	01	01	01
			707/M		0.28	01	01	01	01	01	01	01
			708/M		0.28	01	01	01	01	01	01	01
			709/M		0.29	01	01	01	01	01	01	01
			710/M		0.30	01	01	01	01	01	01	01
ZL0580	Group 3（300 mg/kg. PO）	Female	801/F	10	0.31	01	01	01	01	01	01	01
			802/F		0.32	01	01	01	01	01	01	01
			803/F		0.33	01	01	01	01	01	01	01

		804/F	0.32	01	01	01	01	01	01	01
		805/F	0.30	01	01	01	01	01	01	01
		806/M	0.28	01	01	01	01	01	01	01
		807/M	0.28	01	01	01	01	01	01	01
	Male	808/M	0.28	01	01	01	01	01	01	01
		809/M	0.29	01	01	01	01	01	01	01
		810/M	0.30	01	01	01	01	01	01	01

Number of symptoms

01. normal	02. Cavity to observe	03. Discolored skin	04. Salivation	05. Emesis	06. Lacrimation
07. ptosis	08.Decreased respiration	09. Increased respiration	10. anhelation	11. Ataxia	12. Loose stools
13. Limb's tremor	14. Eye	15.Weakness	16. Hyperreactivity	17. Emaciation	18. Whole body touch
19. Cough	20. At the injection site	21. Alopecia genitalized	22. convulsion	23. Corna	24. Death
Classify:	normal;	slight+	midrange++	severe+++	

Table B. Serum Biochemistry

Parameters Evaluated	Unit	Group 1(Vehicle)															
		Sampling Date: 2020-04-23															
		601F	602F	603F	604F	605F	**Mean**	**Std. Dev.**	**P Value**	606M	607M	608M	609M	610M	**Mean**	**Std. Dev.**	**P Value**
alanine aminotransferase (ALT)	U/L	20.3	26.2	26.4	20.7	51.6	31.23	13.84	0.31	24.8	26.8	31	47.5	60.5	54.00	9.19	0.08
aspartate aminotransferase (AST)	U/L	55.7	77.3	201.2	82.4	130.6	109.44	58.14	0.39	99.7	81.1	108.8	96.5	218.5	157.50	86.27	0.05
γ-Glutamyltransferase(γ-GT)	U/L	<2.0	<2.0	<2.0	<2.0	<2.0	<2.0	NA	NA	<2.0	<2.0	<2.0	<2.0	2.7	2.70	NA	NA
total protein (TP)	g/L	50.4	51.7	53.8	52.8	54.3	52.60	1.58	0.09	54.9	54	52.2	47.6	54.5	51.05	4.88	0.14
albumin (ALB)	g/L	21.8	20.3	21.5	21.5	21.8	21.38	0.62	0.10	22.7	20.6	22.2	19.7	23	21.35	2.33	0.07
globin(GLB)	g/L	28.6	31.4	32.3	31.3	32.5	31.22	1.56	0.08	32.2	33.4	30	27.9	31.5	29.70	2.55	0.28
Albumin/globulin ratio(A/G)	None	0.8	0.6	0.7	0.7	0.7	0.70	0.07	0.31	0.7	0.6	0.7	0.7	0.7	0.70	0.00	0.50
total bilirubin (TBIL)	μmol/L	<0.7	0.9	3.4	<0.7	<0.7	2.15	1.77	NA	<0.7	1.1	<0.7	1.4	1.1	1.25	0.21	NA
alkaline phosphatase (ALP)	U/L	78.9	106.7	110.7	90.8	169.1	111.24	34.76	0.21	163.4	129.1	91.2	120.2	200.7	160.45	56.92	0.05
lactate dehydrogenase(LDH)	U/L	612.8	672.7	898.7	634.2	867.6	737.20	135.40	0.48	1051	695	791	371.4	606.6	489.00	166.31	0.42
UREA	mmol/L	5.3	6.1	10	6.2	5	6.52	2.01	0.22	8.8	9.5	7.5	4.2	5.4	4.80	0.85	0.20
creatinine (Crea)	μmol/L	9.1	10.4	10.7	8.4	8.8	9.48	1.01	0.29	8.6	9.8	10	8.1	9.8	8.95	1.20	0.31
total cholesterol (TCHO)	mmol/L	2.2	5.04	4.7	3.96	2.71	3.72	1.23	0.41	3.65	5.56	2.67	2.24	3.21	2.73	0.69	0.15
triglyceride (TG)	mmol/L	0.95	1.24	0.87	1.64	2.42	1.42	0.63	0.17	0.82	1	1.15	1.03	0.99	1.01	0.03	0.21
glucose (GLU)	mmol/L	11.28	5.49	10.83	5.12	4.55	7.45	3.31	0.09	7.44	12.25	5.15	9.22	8.53	8.88	0.49	0.37
creatine kinase (CK)	U/L	92	115	829	166	350	310.40	307.10	0.39	260	321	292	191	705	448.00	363.45	0.37
T-CA	mmol/L	2.78	2.75	2.92	2.84	2.75	2.81	0.07	0.08	2.85	2.87	2.8	2.72	2.87	2.80	0.11	0.42

PHOS	mmol/L	>3.0	>3.0	>3.0	>3.0	2.05	2.05	NA	NA	>3.0	>3.0	>3.0	>3.0	>3.0	>3.0	NA	NA
K	mmol/L	9	9.7	11.7	10.4	12.4	10.64	1.40	0.45	13.10	14.70	12.30	12.60	13	12.80	0.28	0.40
Na	mmol/L	153	163	156	158	162	158.40	4.16	0.26	161	154	157	154	174	164.00	14.14	0.13
Cl	mmol/L	113	116	115	113	119	115.20	2.49	0.09	119	108	113	107	120	113.50	9.19	0.24

Parameters Evaluated	Unit	Group 2 (100 mg/kg)															
		Sampling Date: 2020-04-23															
		701F	602F	603F	604F	605F	**Mean**	**Std. Dev.**	**P Value**	606M	607M	608M	609M	610M	**Mean**	**Std. Dev.**	**P Value**
alanine aminotransferase (ALT)	U/L	21.7	26.2	26.4	20.7	51.6	31.23	13.84	0.31	24.8	26.8	31	47.5	60.5	54.00	9.19	0.08
aspartate aminotransferase (AST)	U/L	72.8	77.3	201.2	82.4	130.6	109.44	58.14	0.39	99.7	81.1	108.8	96.5	218.5	157.50	86.27	0.05
γ-Glutamyltransferase(γ-GT)	U/L	<2.0	<2.0	<2.0	<2.0	<2.0	<2.0	NA	NA	<2.0	<2.0	<2.0	<2.0	2.7	2.70	NA	NA
total protein (TP)	g/L	58.2	51.7	53.8	52.8	54.3	52.60	1.58	0.09	54.9	54	52.2	47.6	54.5	51.05	4.88	0.14
albumin (ALB)	g/L	23.90	20.3	21.5	21.5	21.8	21.38	0.62	0.10	22.7	20.6	22.2	19.7	23	21.35	2.33	0.07
globin(GLB)	g/L	34.3	31.4	32.3	31.3	32.5	31.22	1.56	0.08	32.2	33.4	30	27.9	31.5	29.70	2.55	0.28
Albumin/globulin ratio(A/G)	None	0.7	0.6	0.7	0.7	0.7	0.70	0.07	0.31	0.7	0.6	0.7	0.7	0.7	0.70	0.00	0.50
total bilirubin (TBIL)	μmol/L	<0.7	0.9	3.4	<0.7	<0.7	2.15	1.77	NA	<0.7	1.1	<0.7	1.4	1.1	1.25	0.21	NA
alkaline phosphatase (ALP)	U/L	74.5	106.7	110.7	90.8	169.1	111.24	34.76	0.21	163.4	129.1	91.2	120.2	200.7	160.45	56.92	0.05
lactate dehydrogenase(LDH)	U/L	798.5	672.7	898.7	634.2	867.6	737.20	135.40	0.48	1051	695	791	371.4	606.6	489.00	166.31	0.42
UREA	mmol/L	5.8	6.1	10	6.2	5	6.52	2.01	0.22	8.8	9.5	7.5	4.2	5.4	4.80	0.85	0.20
creatinine (Crea)	μmol/L	9.4	10.4	10.7	8.4	8.8	9.48	1.01	0.29	8.6	9.8	10	8.1	9.8	8.95	1.20	0.31
total cholesterol (TCHO)	mmol/L	3.33	5.04	4.7	3.96	2.71	3.72	1.23	0.41	3.65	5.56	2.67	2.24	3.21	2.73	0.69	0.15
triglyceride (TG)	mmol/L	1.52	1.24	0.87	1.64	2.42	1.42	0.63	0.17	0.82	1	1.15	1.03	0.99	1.01	0.03	0.21
glucose (GLU)	mmol/L	8.33	5.49	10.83	5.12	4.55	7.45	3.31	0.09	7.44	12.25	5.15	9.22	8.53	8.88	0.49	0.37
creatine kinase (CK)	U/L	138	115	829	166	350	310.40	307.10	0.39	260	321	292	191	705	448.00	363.45	0.37

T-CA	mmol/L	3.04	2.75	2.92	2.84	2.75	2.81	0.07	0.08	2.85	2.87	2.8	2.72	2.87	2.80	0.11	0.42
PHOS	mmol/L	>3.0	>3.0	>3.0	>3.0	2.05	2.05	NA	NA	>3.0	>3.0	>3.0	>3.0	>3.0	>3.0	NA	NA
K	mmol/L	11.30	9.7	11.7	10.4	12.4	10.64	1.40	0.45	13.10	14.70	12.30	12.60	13	12.80	0.28	0.40
Na	mmol/L	155	163	156	158	162	158.40	4.16	0.26	161	154	157	154	174	164.00	14.14	0.13
Cl	mmol/L	111	116	115	113	119	115.20	2.49	0.09	119	108	113	107	120	113.50	9.19	0.24

Parameters Evaluated	Unit	Group 3 (300 mg/kg)															
		Sampling Date: 2020-06-11															
		801F	802F	803F	804F	805F	**Mean**	**Std. Dev.**	**P Value**	806M	807M	808M	809M	810M	**Mean**	**Std. Dev.**	**P Value**
alanine aminotransferase (ALT)	U/L	35.7	33.8	29.4	32.2	40.2	34.26	4.04	0.27	53.3	42.6	32.1	25.1	37	38.02	10.69	0.21
aspartate aminotransferase (AST)	U/L	155.7	77.9	83.1	103.5	101.7	104.38	30.80	0.26	81.3	173.4	55.7	76.8	90.1	95.46	45.37	0.20
γ-Glutamyltransferase(γ-GT)	U/L	<2.0	<2.0	<2.0	<2.0	<2.0	<2.0	NA	NA	<2.0	<2.0	<2.0	<2.0	<2.0	<2.0	NA	NA
total protein (TP)	g/L	59.4	56.4	51.7	54.4	59.7	56.32	3.39	0.43	49.8	54.4	51.1	49.2	49.5	50.80	2.14	0.48
albumin (ALB)	g/L	24.9	22.8	21.6	20.5	22.6	22.48	1.63	0.47	18.2	22.8	18.1	17.7	18.6	19.08	2.10	0.12
globin(GLB)	g/L	34.5	33.6	30.1	33.9	37.1	33.84	2.51	0.36	31.6	31.6	33	31.5	30.9	31.72	0.77	0.01
Albumin/globulin ratio(A/G)	None	0.7	0.7	0.7	0.6	0.6	0.66	0.05	0.19	0.6	0.7	0.5	0.6	0.6	0.60	0.07	0.01
total bilirubin (TBIL)	μmol/L	<0.7	1.6	1	1.4	<0.7	<0.7	NA	NA	<0.7	<0.7	<0.7	<0.7	1.3	<0.7	NA	NA
alkaline phosphatase (ALP)	U/L	54.9	135.5	101.7	63.4	82.3	87.56	32.31	0.02	117.1	125.4	89.8	131	125.2	117.70	16.36	0.31
lactate dehydrogenase(LDH)	U/L	2079	486.2	541.1	835.5	840.3	956.42	648.44	0.32	781.1	1676.7	329.1	1142.8	732.7	932.48	506.21	0.21
UREA	mmol/L	7.5	6.4	6.7	5.6	7.2	6.68	0.74	0.22	8.8	10.8	7	5.2	6.7	7.70	2.15	0.14
creatinine (Crea)	μmol/L	<1.50	8.6	6.6	5.9	6.1	6.80	1.24	0.04	17	<1.50	8.1	6.9	9.5	10.38	4.54	0.38
total cholesterol (TCHO)	mmol/L	3.93	3.31	2.43	3.17	3.03	3.17	0.54	0.27	3.78	5.31	4.91	5.56	4.24	4.76	0.74	0.05
triglyceride (TG)	mmol/L	1.56	1.12	1.16	1.75	1.4	1.40	0.27	0.00	1.15	1.92	1.52	2.2	1.17	1.59	0.46	0.11
glucose (GLU)	mmol/L	4.71	5.8	8.69	4.51	8.74	6.49	2.09	0.04	14.31	5.85	9.57	15.59	4.68	10.00	4.89	0.16

creatine kinase (CK)	U/L	135	126	151	171	179	152.40	22.67	0.02	158	132	78	180	313	172.20	87.43	0.17
T-CA	mmol/L	1.47	2.59	2.52	2.67	3.17	2.48	0.62	0.06	3.18	1.62	2.52	2.55	2.43	2.46	0.56	0.08
PHOS	mmol/L	4.38	3.66	3.65	4.44	4.39	>3.0	NA	NA	4.66	4.56	3.73	3.85	3.92	>3.0	NA	NA
K	mmol/L	7.7	10	9.2	9.7	10.5H	9.15	1.02	0.05	10.1H	7.9	12.2H	10.4H	13.1H	7.90	NA	NA
Na	mmol/L	160	157	157	156	158	157.60	1.52	0.23	157	161	153	157	154	156.40	3.13	0.32
Cl	mmol/L	111	109	111	107	110	109.60	1.67	0.02	106	105	106	104	110	106.20	2.28	0.00

If $P < 0.05$, there is significant di`erence; if $P < 0.01$, there is very significant di`erence.

Parameters Evaluated	Unit	Group 4 (control)														
		Sampling Date: 2020-04-23														
		801F*	802F*	803F*	804F*	805F*	Mean	Std. Dev.	806M*	807M*	808M*	809M*	810M*	Mean	Std. Dev.
alanine aminotransferase (ALT)	U/L	41.8	52.6	20.2	35.7	36.7	37.40	11.72	30.3	18.7	25.6	40.2	41.3	31.22	9.64
aspartate aminotransferase (AST)	U/L	95.5	130.1	96	130.9	139	118.30	20.88	86.5	51.5	62.3	78.5	83.6	72.48	15.00
γ-Glutamyltransferase(γ-GT)	U/L	<2.0	<2.0	<2.0	<2.0	<2.0	<2.0	NA	<2.0	<2.0	<2.0	<2.0	<2.0	<2.0	NA
total protein (TP)	g/L	49.6	59.6	53.2	54.5	62.3	55.84	5.09	50	49.9	50.4	51.5	51.9	50.74	0.91
albumin (ALB)	g/L	21.1	23.8	21.5	22.1	24.3	22.56	1.42	20.2	20.2	19.7	20.6	21.7	20.48	0.75
globin(GLB)	g/L	28.5	35.8	31.7	32.4	38	33.28	3.70	29.8	29.7	30.7	30.9	30.2	30.26	0.53
Albumin/globulin ratio(A/G)	None	0.7	0.7	0.7	0.7	0.6	0.68	0.04	0.7	0.7	0.6	0.7	0.7	0.68	0.04
total bilirubin (TBIL)	μmol/L	<0.7	<0.7	<0.7	<0.7	<0.7	<0.7	NA	<0.7	2.4	<0.7	<0.7	<0.7	<0.7	NA
alkaline phosphatase (ALP)	U/L	111.9	124.5	145.7	130.6	119.2	126.38	12.80	111.9	105.7	106.4	100.8	139.3	112.82	15.32
lactate dehydrogenase(LDH)	U/L	337.8	1293.7	636.6	636.7	828.9	746.74	352.63	792.3	665.2	691.5	840.8	662.1	730.38	81.32
UREA	mmol/L	6	9.8	7.4	7.1	6.7	7.40	1.44	6.1	7.8	7.1	5.2	7.4	6.72	1.06
creatinine (Crea)	μmol/L	10.5	8.9	10.7	11.4	8.3	9.96	1.30	9.1	8.6	9.4	9.1	10.6	9.36	0.75
total cholesterol (TCHO)	mmol/L	1.98	5.19	3.75	3.29	3.94	3.63	1.16	4.48	3.55	3.83	4.74	2.05	3.73	1.05
triglyceride (TG)	mmol/L	1.21	0.96	0.96	1.42	1.22	1.15	0.20	1.37	1.36	0.85	1.98	1.18	1.35	0.41
glucose (GLU)	mmol/L	10.09	14.01	9.42	12.64	8.27	10.89	2.37	11.31	12.92	11.78	17.2	5.82	11.81	4.07
creatine kinase (CK)	U/L	194	319	154	291	365	264.60	87.97	209	70	96	137	124	127.20	52.54
T-CA	mmol/L	2.79	3.16	2.84	3.01	3.01	2.96	0.15	2.76	2.9	2.71	3.21	3.15	2.95	0.23

PHOS	mmol/L	>3.0	>3.0	>3.0	>3.0	>3.0	>3.0	NA	>3.0	>3.0	>3.0	>3.0	>3.0	>3.0	NA
K	mmol/L	10.10	13.10	8.9	11.50	8.7	10.46	1.85	12.70	15.80	12.20	12.20	12.40	13.06	1.55
Na	mmol/L	157	153	157	159	157	156.60	2.19	156	155	155	155	157	155.60	0.89
Cl	mmol/L	114	113	112	113	111	112.60	1.14	113	113	112	108	112	111.60	2.07

Table C. The result of necropsy macroscopic after oral dose of ZL0580 at 100, 300 mg/kg dose levels.

Compound	Group (Dose level)	Gender	Animal No.	Necropsy Macroscopic															
				**Classify: normal-; slight +; midrange ++; severe ++++**															
				skin	hair	lips	mouth	heart	spleen	lung	thymus	kidney	adrenal gland	pancreas	gastrointestinal tract	testis	epididymis	uterus	ovary
Vehicle	Group 1（0 mg/kg. PO）	Female	601/F	–	–	–	–	–	–	–	–	–	–	–	–	NA	NA	–	–
			602/F	–	–	–	–	–	–	–	–	–	–	–	–	NA	NA	–	–
			603/F	–	–	–	–	–	–	–	–	–	–	–	–	NA	NA	–	–
			604/F	–	–	–	–	–	–	–	–	–	–	–	–	NA	NA	–	–
			605/F	–	–	–	–	–	–	–	–	–	–	–	–	NA	NA	–	–
		Male	606/M	–	–	–	–	–	–	–	–	–	–	–	–	–	–	NA	NA
			607/M	–	–	–	–	–	–	–	–	–	–	–	–	–	–	NA	NA
			608/M	–	–	–	–	–	–	–	–	–	–	–	–	–	–	NA	NA
			609/M	–	–	–	–	–	–	–	–	–	–	–	–	–	–	NA	NA
			610/M	–	–	–	–	–	–	–	–	–	–	–	–	–	–	NA	NA
ZL0580	Group 2（100 mg/kg. PO）	Female	701/F	–	–	–	–	–	–	–	–	–	–	–	–	NA	NA	–	–
			702/F	–	–	–	–	–	–	–	–	–	–	–	–	NA	NA	–	–
			703/F	–	–	–	–	–	–	–	–	–	–	–	–	NA	NA	–	–
			704/F	–	–	–	–	–	–	–	–	–	–	–	–	NA	NA	–	–
			705/F	–	–	–	–	–	–	–	–	–	–	–	–	NA	NA	–	–
		Male	706/M	–	–	–	–	–	–	–	–	–	–	–	–	–	–	NA	NA
			707/M	–	–	–	–	–	–	–	–	–	–	–	–	–	–	NA	NA
			708/M	–	–	–	–	–	–	–	–	–	–	–	–	–	–	NA	NA
			709/M	–	–	–	–	–	–	–	–	–	–	–	–	–	–	NA	NA
			710/M	–	–	–	–	–	–	–	–	–	–	–	–	–	–	NA	NA
ZL0580	Group 3（300 mg/kg. PO）	Female	801/F	–	–	–	–	–	–	–	–	–	–	–	–	NA	NA	–	–
			802/F	–	–	–	–	–	–	–	–	–	–	–	–	NA	NA	–	–
			803/F	–	–	–	–	–	–	–	–	–	–	–	–	NA	NA	–	–
			804/F	–	–	–	–	–	–	–	–	–	–	–	–	NA	NA	–	–
			805/F	–	–	–	–	–	–	–	–	–	–	–	–	NA	NA	–	–

Male	806/M	–	–	–	–	–	–	–	–	–	–	–	–	–	–	NA	NA
	807/M	–	–	–	–	–	–	–	–	–	–	–	–	–	–	NA	NA
	808/M	–	–	–	–	–	–	–	–	–	–	–	–	–	–	NA	NA
	809/M	–	–	–	–	–	–	–	–	–	–	–	–	–	–	NA	NA
	810/M	–	–	–	–	–	–	–	–	–	–	–	–	–	–	NA	NA

NA: Not applicable.

Table D: List of primers used in RT-qPCR

S. No.	Gene name	Oligo's	Sequences (5'-3')
1	HMGCS1	Forword	CTCCCTGACGTGGAATGTCT
		Reverse	GAACTGTCTGCCCAGGTGAT
2	SQLE	Forword	GGCATTGCCACTTTCACCTAT
		Reverse	GGCCTGAGAGAATATCCGAGAAG
3	DHCR7	Forword	GCTGCAAAATCGCAACCCAA
		Reverse	GCTCGCCAGTGAAAACCAGT
4	ASS1	Forword	TCCGTGGTTCTGGCCTACA
		Reverse	GGCTTCCTCGAAGTCTTCCTT
5	ITK	Forword	GAAGATCGTCATGGGAAGAAGC
		Reverse	CGGGTATTTATAGTGGCATGGG
6	DUSP8	Forword	TCAGCTCCGTCAACATCTGC
		Reverse	CGCGTGCTCTGGTCATAGA
7	KLF10	Forword	CTTCCGGGAACACCTGATTTT
		Reverse	GCAATGTGAGGTTTGGCAGTATC
8	WDR52	Forword	CCAACCTACAACTTCTGGACG
		Reverse	CACTGCTACTTCGCAGGTAGA
9	GAPDH	Forword	GGCCTCCAAGGAGTAAGACC
		Reverse	AGGGGTCTACATGGCAACTG

Table E: Primer sequences for ChIP-qPCR

Gene name	Oligo's	Sequences (5'-3')
5' LTR (HIV)	Forword	GTTAGACCAGATCTGAGCCT
	Reverse	GTGGGTTCCCTAGTTAGCCA
